# Extended pesticide soil monitoring in Cuban potato (*Solanum tuberosum* L.) production: residue co-occurrence, dissipation rates, ecological risks, and implications

**DOI:** 10.1039/d5em00119f

**Published:** 2025-08-13

**Authors:** Brizeidi Peña, Isabel Hilber, Dayana Sosa, Arturo C. Escobar, Thomas D. Bucheli

**Affiliations:** a Centro Nacional de Sanidad Agropecuaria (CENSA), Unidad Analítica de Residuos y Contaminantes Apartado 10 CP 32700 San José de las Lajas Mayabeque Cuba dayanasosap@gmail.com; b Agroscope Environmental Analytics Reckenholzstrasse 191 8046 Zurich Switzerland thomas.bucheli@agroscope.admin.ch +41 58 468 7342

## Abstract

Pesticides are intensively used but understudied in tropical regions in America. We therefore investigated their occurrence and dissipation in soils of 18 potato (*Solanum tuberosum* L.) producing farms in Mayabeque, Cuba, between 2018 and 2022. Between two and 17 active ingredients (AIs) were used per site, and the cultivation period and sums of AIs ranged from 0.001 to 26 kg_AI_ ha^−1^. Soil concentrations of 38 individual target compounds ranged from 0.1 to 658 μg kg_dry weight_^−1^. Observed half-lives (DT_50,obs_) of the five most prevalent AIs were up to eight times lower than the DT_50_ from temperate climate in the Pesticides Properties Database. The fate and behaviour of pesticides rather depended on their physico-chemical than on soil properties. Several sites posed a high risk to earthworms (cumulative risk quotient >1) during periods of peak pesticide application to harvest, with azoxystrobin and cyproconazole contributing the most.

## Abstract 2

While our extended pesticide soil monitoring under potato cultivation confirms generally shorter half-lives under tropical conditions, the corresponding residues might still pose a risk to terrestrial ecosystems.

## Graphical abstract

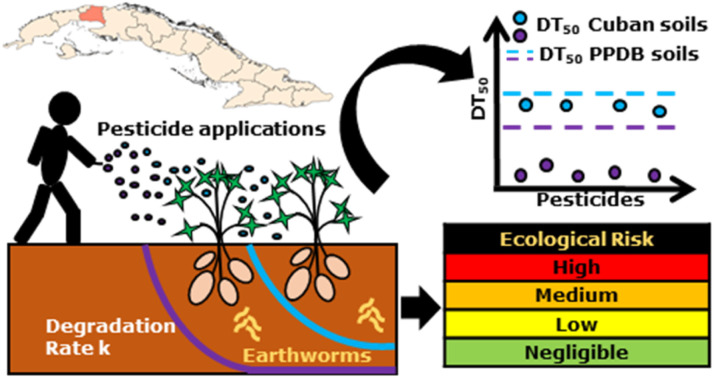

**Environmental significance**

Applications of modern pesticides is vital to ensure crop production worldwide, but particularly under tropical conditions. Their environmental risk assessment is still mainly based on data from temperate regions, which are hardly representative of conditions prevailing in the global south. Pesticide application records and their residues over time are reported for the first time in Cuban soils under conventional potato production. Degradation was considerably faster than in temperate soils for most of the applied pesticides, but residues still displayed a potential chronic ecotoxicological risk in many cases. With this study, we provide data to validate conceptual and modelling approaches on pesticide fate and behaviour in the tropics and set the ground for the establishment of locally adapted regulatory guidance values.

## Introduction

Pesticides are used worldwide to increase food production and reduce yield losses caused by diseases, pests, and weeds.^1^ Pests in tropical areas can be more aggressive than in temperate climates, and pesticides therefore have a lower efficiency.^2^ This could be due to the high relative humidity, heavy rainfall, and year-round high temperatures compared to temperate climates, which can markedly increase the degradation^3^ and distribution of pesticides.^4,5^

Soil monitoring studies are prerequisites for sustainable soil and land management, as, *e.g.*, stipulated by the Sustainable Development Goals, the European Soil Deal,^6^ the “farm to fork” strategy launched in the European Union^7^ (actions ended 2023) or the “Soil-Food-Environment-Health nexus”.^8^ Articles on the monitoring of pesticides in soil have been published from various regions of the world,^9,10^ including South America,^11–13^ Africa,^5,14,15^ Asia,^16,17^ and Europe.^18–22^

Regulatory guidance values (RGVs) related to soil pollution with pesticides vary highly.^23–25^ With regard to pesticides and their environmental risks, places such as North America, Europe, Japan, and Australia established legal frameworks, while requirements, such as soil monitoring programs, in tropical countries are either not available, unclear or inadequately implemented.^4^ The little data available on the fate and even more limited data on the effects of active ingredients (AIs) and transformation products (TPs) of tropical areas do not allow databases to be established or risks to be assessed that are tailored to this temperature, for example, for soil-dwelling organisms,^26^ let alone RGVs for tropical soils to be recommended.

Cuba makes a special case in many respects. Data of pesticides in the Cuban environment are scarce. In 1996, the presence of eleven legacy compounds in soil was published.^27^ Only recently, preliminary data became available due to an analytical method development for pesticides in Cuban soils.^28^*Solanum tuberosum* L. (potato) is an important crop worldwide, and pesticide use is intensive.^29^ Although Cuba has limited quantities of pesticides available,^30^ potato is the most prioritized crop in the country. About 52 AIs were applied between 2011 and 2013, which correspond to 2–6 kg_AI_ ha^−1^ per year.^30^ The Food and Agriculture Organisation (FAO) estimated for Cuba around 1 kg_AI_ ha^−1^ per year during the same period and 0.75 kg_AI_ ha^−1^ in 2022. In comparison, the FAO reported 12 kg_AI_ ha^−1^ for Brasil, 17 kg_AI_ ha^−1^ for Costa Rica, 27 kg_AI_ ha^−1^ in Saint Lucia, and 3 kg_AI_ ha^−1^ in the USA in 2022.

Due to the data gap in Cuba regarding the monitoring of pesticide residues in agricultural soils and the importance of potato, and also for comparison with other tropical or South American countries, a monitoring program was established and conducted. Unlike many other studies cited throughout this paper, this project monitored a larger number of commonly used pesticides in soils from different farms over four consecutive years at three sampling times per cultivation period (CP) in Mayabeque, Cuba. Sampling took place during wet and dry seasons, accounting for different soil moisture conditions and plant growth stages in accordance with recommendations from the literature.^4,5^ Objectives were (i) to determine AI and TP residues in soils from different potato fields of 18 farms from 2018 to 2022, (ii) to calculate the dissipation rate of some AIs on the basis of predicted environmental concentrations in soil (PEC_soil_ or *A*_0_) and compare the observed half-lives (DT_50,obs_) with the literature and a database, (iii) relate the DT_50,obs_ to soils' and pesticides' physico-chemical properties, and (iv) to calculate risk quotients (RQs) arising from pesticide residues in soils for earthworms (*Eisenia fetida*).

With this extended monitoring of pesticide residues resulting from repeated applications under realistic agricultural conditions, we aim to provide data to validate conceptual and modelling approaches on pesticide fate and behavior in the tropics.^3,4,31^ In addition to this article, the data will serve as a basis and to foster monitoring databases of Cuba and the Latin American network,^32^ to build consistent RGVs for tropical soils. Finally, it delivers added value of information for stakeholders of different levels of the agricultural production chain such as farmers, pesticide producers, regulatory bodies, as well as consumers not only in Cuba but also in the Caribbean and other tropical areas.

## Materials and methods

### Study area and soil sampling

The study was carried out in the Mayabeque Province (SI, Fig. S1), one of the major potato production regions in Cuba (*e.g.*, 29.2 mil tons in 2021,^33^ representing 30% of the total Cuban potato production).^34^ Three municipalities of Mayabeque where potatoes are intensively produced were selected: Batabanó (BT), Quivicán (QV) and San José de las Lajas (SJ). Pesticide application records from farmers managing the sites were collected in 21 sites (Fig. S1). Eighteen conventionally managed sites (S) were sampled in BT (*n* = 8, Table S1, numbers 1 to 8), QV (*n* = 8, numbers 9 to 16) and SJ (*n* = 2, numbers 17 and 18), as well as one organically managed site (Sorg) in SJ (*n* = 1, number 19) and two forest control sites (Scont) in BT (*n* = 1, number 20) and SJ (*n* = 1, number 21). Potato is predominantly produced on rhodic ferralitic nitisol, and all sites belonged to this soil type (Fig. S1). Nitisols are the most productive soils of the humid tropics,^35^ which is why all the farms participating in this study were located on areas of this soil type. Sampling was carried out in four consecutive cultivation periods (2018–2019 (CP1), 2019–2020 (CP2), 2020–2021 (CP3) and 2021–2022 (CP4)) at three time points per growing season: (s1): before planting potato (October–November), (s2): during peak pesticide application (December–January), and (s3): at harvest (March–April). A total of 150 soil samples were collected over the four years in the middle of each field, according to the protocol by the Swiss soil monitoring network (NABO).^36^ In a 10 m × 10 m area, 100 soil cores (one from each m^2^) were taken with an auger of 1.8 cm diameter at a soil depth of 0–20 cm. Pesticide soil monitoring often focuses on this layer (*e.g.*, Froger *et al.*,^37^ Silva *et al.*,^20^ and Riedo *et al.*^38^), as concentrations often decrease with depth (*e.g.*, Barmettler *et al.*^39^ and Mangold *et al.*^40^). Four times 25 cores were united to four composite samples per 100 m^2^, immediately brought to the lab, dried in an air-conditioned room of 25 °C at 5% humidity for seven days until constant weight, crushed and sieved over a 2 mm sieve and stored in the dark at ambient temperature. After extended manual shaking of one of the four composite samples, a subsample of 100 g was gathered before shipping to Agroscope, Switzerland, for analysis. Soil analysis, such as bulk density (bd), organic carbon (OC) content, pH, and texture, and basal respiration (BR) and microbial biomass (MB) determination are described in the SI, chapter S1 and Table S2.

### Extraction, detection and quantification of pesticide residues in soils

Pesticides for analysis (Table S3) were selected according to the criteria explained in Peña *et al.*^28^ Briefly, pesticides were chosen on the basis of official lists of pesticide application to potato crop in Cuba from 2008, 2016, and 2022.^41–43^ Also, legacy compounds, such as endosulfans (α and β) and their TP endosulfan sulphate, were included, because although banned in Cuba since 2013 ^30^ and therefore removed from the official lists of 2016 ^42^ and 2022,^43^ they remained in use. Thirty-one AIs (48% were fungicides (F), 29% were herbicides (H), and 23% were insecticides (I)) and seven TPs were quantified. Physico-chemical properties of compounds including DT_50,field_ reported in the PPDB^44^ that served as reference values are shown in Table S3. Note that the origin, type and degree of data aggregation in the PPDB is only partially disclosed.^44^

The Quick, Easy, Cheap, Effective, Robust and Safe (QuEChERS) method validated by Peña *et al.*^28^ served to extract the analytes from the soil samples. Briefly, 5 g of dried soil with 10 ng g_soil_^−1^ isotopically labelled internal standard (IL-IS) mixture was extracted with 5 mL Milli-Q and 5 mL acetonitrile containing 2.5% formic acid. To clean up the extract, 2 g MgSO_4_, 0.5 g NaCl, and 0.5 g CH_3_COONa were added, vortexed, homogenized (TURBULA, Willy A. Bachofen AG, Muttenz, Switzerland), sonicated (Sonorex Digital 10 P, Bandelin from IG, Zurich, Switzerland), and centrifuged (Eppendorf, Hamburg, Germany). One mL of supernatant extract was filtered through a 0.20 μm Chromafil® PET filter. Finally, 10 ng g_soil_^−1^ syringe standard (triphenyl phosphate) was added. The extract was directly injected into a gas chromatography coupled to tandem mass spectrometry (GC-MS/MS; MS-TQ8050 NX with GC-2030, Shimadzu Corp., Kyoto, Japan) system for analysis. Details about the method and the optimized GC-MS/MS conditions can be found in Peña *et al.*^28^ Quality control and assurance were described by and performed according to Peña *et al.*^28^

### Pesticide application records of farmers and application – detection scenarios

Records of applied pesticides per farmer/site were collected between 2018 and 2021. Application records were not available for 2022 (CP4), which is why the chapters “application–detection scenarios” and “observed half-lives of frequently applied and detected pesticides in Cuban soils” refer to the first three CPs only. Pesticides were applied at all conventional sites (*n* = 18). In contrast, pesticides were never applied at the organically managed site (*n* = 1), according to farmers' records over the last 10 years.

Application–detection scenarios were compared for all analyzed compounds (31 AIs and seven TPs) at 19 sites over three consecutive CPs, CP1–3, with 38 incidences in total (note that not all sites were sampled in all CPs and at all timepoints, Table S1). Therefore, for each compound, site and CP, repeated applications were summed up, and median concentrations in the three soil samples collected at different timepoints (s1–s3) were calculated. This resulted in 1178 application–detection cases for AIs, which were then separated into four categories following the procedure of Chiaia-Hernandez *et al.*,^18^*i.e.*, (i) pesticide applied at a given site according to the farmers' record and detected in the corresponding soil sample (true-positive), (ii) pesticide applied but not detected (false-negative), (iii) pesticide not applied but detected (false-positive), and (iv) pesticide not applied and not detected (true-negative). The detections of TPs were related to the application of their parents, which resulted in 266 cases. Control sites were not included in the scenarios.

### Data analysis

Data were statistically described and evaluated with R version 4.3.2 (2023-10-31 ucrt). Firstly, compound residues detected were evaluated with the non-linear mixed effect model (nlme). It allows the division of independent variables into factors of fixed effects (CP, s1–3, and type of residue found) and random effects, which is the site (Table S4). The influence of random effects is not accounted for in the model because each site was repeatedly sampled per CP, and sampling time and measurements are therefore not independent from each other. This approach corresponds to a split block design that additionally accounts for the fact that not all sites could be sampled over four consecutive years. The dependent variable soil concentration was transformed by logarithmization, whereby no detects or concentrations below the limit of quantification (LOQ) were omitted. The nlme model also served for the evaluation of single compounds most frequently detected, where the logarithmized concentration was the dependent variable and sampling time was the independent variable with fixed effects and again site with random effects (Table S5). The factor CP was deliberately omitted in this evaluation because application or availability might have been overruled by economic constraints rather than infestation of pests. If the sampling time had a significant influence on the nlme model, a pairwise comparison of levels with the Student's *t*-test was run, where the *p*-value was adjusted according to Bonferroni as conservative correction accounting for multiple testing.

Secondly, for pesticides with frequent true-positive cases (ametryn, azoxystrobin, chlorothalonil, cyproconazole, and *S*-metolachlor), residues were quantitatively evaluated in combination with application records (2018–2021) to evaluate their persistence in Cuban soils. Soil concentration after application was predicted (*A*_0_; [μg kg_dry weight (dw)_^−1^], eqn (1)) considering the application rate (App; [g ha^−1^]) of an AI, the depth (*d*, 20 [cm]) and bd [g cm^−3^] of the soil and the crop interception (*f*_int_, [−]) at the time of application according to the FOCUS workgroup.^45^ Crop interception was 0 for pre-emerging H, *e.g.*, ametryn and *S*-metolachlor, because they were applied between 0 and 15 days after planting the potato tubers when the soil was still fully exposed to the AI. In contrast, *f*_int_ was considered 0.5 when F was applied after approximately 40–89 days because flowering of the potato intercepted the way to the soil by about 50% plant coverage.1
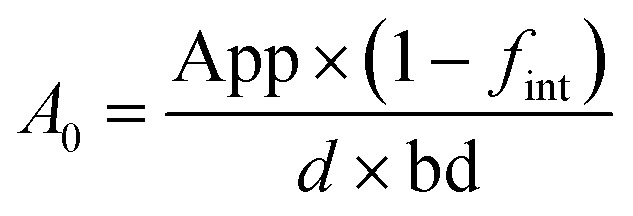


Half-lives of AI were estimated from the application *A*_0_ and the measured concentrations as follows:

AIs that were applied only once per CP (*i.e.*, ametryn and *S*-metolachlor) or that did not show residues of preceding applications were fitted with a single first order dissipation model (eqn (2)), where the metabolic capacity of the degrading biomass was expected to never be constrained.^46^

2 *C*_soil,det_ = *A*_0_ × e^−*kt*^

Pesticides that were repeatedly applied between two sampling timepoints (*i.e.*, azoxystrobin, chlorothalonil, and cyproconazole) were fitted with a sequential single first-order dissipation model as follows (eqn (3a)–(3d))

3a *C*_1_ = *A*_0_e^−*k*(*t*_1_ − *t*_0_)^ + *A*_1_

3b *C*_2_ = *C*_1_e^−*k*(*t*_2_ − *t*_1_)^ + *A*_2_

3c *C*_soil,det_ = *C*_2_e^−*k*(*t*_3_ − *t*_2_)^

Replacing *C*_2_ and *C*_1_ with the right side of the respective equation resulted in

3d *C*_soil,det_ = e^−*kt*_3_^(*A*_0_ + *A*_1_e^*kt*_1_^ + *A*_2_e^*kt*_2_)^

where *C*_soil,det_ is the detected concentration at a given sampling time. The concentration [μg kg_dw_^−1^] in soil (*C*_1_, eqn (3a)) at the second application is the result of the first (*A*_0_, eqn (1)) degraded according to *k* [1 per day] between *t*_1_ and *t*_0_ (where *t* is time [days] and *t*_0_ is 0) and the amount applied *A*_1_ [μg kg_dw_^−1^]. Soil concentration *C*_2_ (eqn (3b)) at the third application depends on the AI that degraded between *t*_2_ and *t*_1_ and the amount applied *A*_2_, *etc.*Eqn (3d) could be adapted to more or less applications and was solved numerically in *R* to find a *k* for a specific farm/site and sequential application. With this *k*, the DT_50,obs_ of the AIs in soil was derived (eqn (4)).4
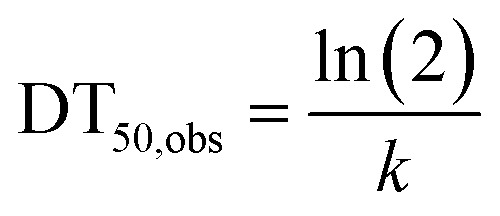


After an application, the DT_50,obs_ reflected the half-life from an application to the next sampling time, *e.g.*, for pre-emergence H, it is s2. If a lower concentration of the same application was detected in s3, a new DT_50,obs_ was also calculated according to the residues in s2 and s3 and the time elapsed in between. The residue at s2 was then considered a pseudo-application as well as, for instance, s3, of which concentrations were detected in s1.

Thirdly, to discern the influence of the factors physico-chemical properties of the compounds and their sum of detected concentrations and application amounts on the observations, AIs and TPs, principal component analysis (PCA) was performed. PCA reduces the dimensions, where the first two principal components (PC) can be depicted in a two-dimensional plot (biplot), but the variance of the multi-dimensional data is as representative as possible.

Fourthly, soil properties, such as bd, OC, soil texture, and pH, and the microbial activity (BR and MB) and the DT_50,obs_ of ametryn, azoxystrobin, chlorothalonil, cyproconazole, and *S*-metolachlor were (cor)related and depicted in pairs plots per compound. While the lower half of the pairs plot depicts the scatter plot, the upper half shows correlation coefficients (|*r*|). For a better overview, the number size correlates positively with |*r*|.

### Chronic risk quotient calculations for earthworms in soil

Chronic risks posed by pesticide residues in soil were calculated based on the risk quotient (RQ, eqn (5)).^47,48^ Toxicological data of *E. fetida* were used, because it is a key species in terrestrial ecosystems, and data are available for most pesticides in the PPDB.^44^5
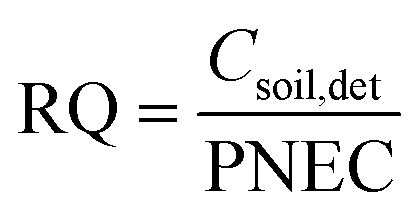
where *C*_soil,det_ is the pesticide concentration detected in soil. The predicted non-effect concentration (PNEC) was derived from eqn (6):6
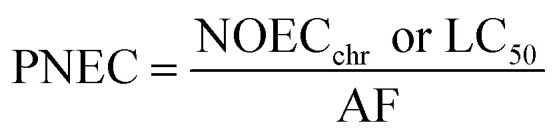


The NOEC_chr_ (eqn (6)) was the chronic no-observed effect concentration in *E. fetida* for reproduction^49^ according to PPDB^44^ data (Table S6). In case the NOEC_chr_ was missing, the lethal concentration that kills half of the population (LC_50_) was taken.^44,50^ Assessment factor (AF) values were established according to the criterion proposed by Suh *et al.*,^47^ Hagner *et al.*,^48^ and Pelosi *et al.*,^49^ who applied them to earthworms. If the NOEC_chr_ for a compound was available, the AF value was 10. In case the NOEC_chr_ was absent and only LC_50_ was indicated, an AF of 1000 was used. As the calculation of RQ was based on earthworm toxicological data only, criteria concerning other trophic levels were not applied.^51^ The sum of RQ over all pesticides (*Σ*RQ) was calculated using eqn (7)7
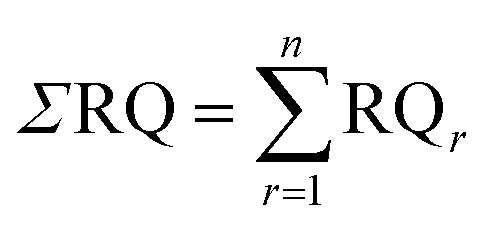
where RQ_*r*_ is the risk quotient for the *r*^th^ AI or TP quantified at a given site, CP, and sampling time.

## Results & discussion

### Monitoring pesticide residues and transformation products in soils of potato crops in Mayabeque, Cuba

Out of a total of 150 samples over four CPs, 84% (126 out of 150) of the samples contained F (Fig. S2, sum (*Σ*_minimum(min) − maximum(max)_): 1.2–423 μg kg_dw_^−1^; median: 30 μg kg_dw_^−1^), 83% H (*Σ*_min − max_: 0.3–688 μg kg_dw_^−1^; median: 25 μg kg_dw_^−1^), 73% I (*Σ*_min − max_: 0.1–35 μg kg_dw_^−1^; median: 4.2 μg kg_dw_^−1^), and 47% TP (*Σ*_min − max_: 0.1–210 μg kg_dw_^−1^; median: 7.0 μg kg_dw_^−1^). The nlme model (Fig. S3 and Table S4) revealed highly significant (*p*-value <0.0001) influences of the sampling time, type of compound, and CP. However, when the concentrations were compared amongst CPs, no significant difference was discernible. This result is plausible because pesticide residues in soil primarily depend on the timepoint when the AI was applied with respect to the crop growth, the amount, the pest or disease incidence, and when the soil was sampled, but rather not on the year or CP. Sampling s1 is understood as background sampling, which is why s1 showed minimum sums at most sites. Exceptions to this rule are S02 and S03 in CP4 (Fig. S2), where *S*-metolachlor might have been applied to corn, a preceding crop, before s1, and S04 in CP2, where azoxystrobin might have been used to protect, for example, vegetables (Tables S1 and S7). Residue types differed significantly from each other except F and H (Fig. S3).

**Fig. 1:**
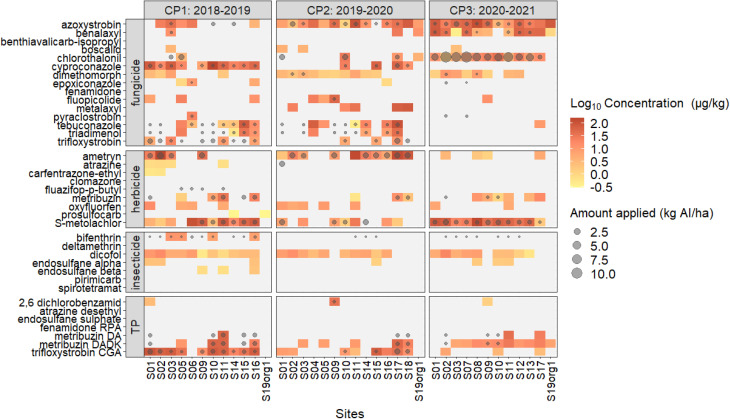
Relationship between pesticides applied, analyzed, and detected in Cuban soil under potato cultivation. Grey points represent the sum of active ingredients (AIs) applied (kg_AI_ ha^−1^) per cultivation period (CP1–CP3) and site (S1–S19). The higher the amount of AI applied, the larger the grey circle. Median of logarithmized concentrations of pesticides detected above the limit of quantification (LOQ) in soil samples (μg kg^−1^) are shown in the heatmap as yellow to deep red cells. Light grey cells indicate concentrations below the LOQ. Coincidence between coloured cells and grey circles indicate a true positive case (applied–detected). Empty cells indicate true-negative cases (not applied–not detected). Coloured cells without a grey circle show false-positives (not applied but detected) and cells with a grey circle only are false-negatives (applied but not detected). Transformation product (TP) scenarios relate to the respective applications (grey circle) of the parent compound. Org: organic potato production.

Azoxystrobin, dicofol, dimethomorph, *S*-metolachlor, and trifloxystrobin CGA were detected in >30% of the 150 samples and therefore statistically evaluated (Table S5 and Fig. S4). Azoxystrobin (F) was found in 69% of the samples and had min and max concentrations of 0.2 and 294 μg kg_dw_^−1^, respectively, (median, 15 μg kg_dw_^−1^). Its concentrations were significantly higher in s3 in comparison to s1, which is in line with its multiple applications between s2 and s3. Similar concentration ranges (1.0 to 328 μg kg^−1^) and frequencies (28% from 110 samples) of azoxystrobin were found in Turkey, although under different temporal and spatial sampling regimes.^52^ Dicofol (I) was detected in 71% of soil samples, and concentrations ranged from 0.1 to 35 μg kg_dw_^−1^ with a median of 3.8 μg kg_dw_^−1^. No differences in residues were found between the sampling times, which is plausible because dicofol was never applied (Fig. 1) during the study period. A similar concentration range (4.3 to 25 μg kg_dw_^−1^) was reported for agricultural soils (with an identical sampling depth) of Senegal^53^ and was even found in some humid, sub-tropical Chinese mountain forest soils at concentrations of up to 50 μg kg_dw_^−1^.^54^ Although forest soils were sampled in this study (Scont), dicofol was not found at these remote sites. Dimethomorph (F) was detected in 45% of the soil samples at concentrations from 1.0 to 72 μg kg_dw_^−1^ and with a median of 2.2 μg kg_dw_^−1^. Concentrations were not influenced by the sampling time. The AI was clearly less frequently detected in the Turkish study with 5.5% than in the present one, but if, concentrations were higher (4.0–862 μg kg^−1^; mean: 134 μg kg^−1^).^52^*S*-Metolachlor (H) was detected in 72% of the samples and ranged from 1.1 to 658 μg kg_dw_^−1^ (median: 17 μg kg_dw_^−1^). As this pre-emergence H was applied before s2, its concentrations were significantly higher in s2 and even s3 than in s1. Reported soil concentrations of *S*-metolachlor in other countries differ from this study. While investigations in France^37^ (0.7–20 μg kg_dw_^−1^; median: 3.3 μg kg_dw_^−1^) and Switzerland^21^ (0.3–79 μg kg_dw_^−1^; median: 2.5 μg kg_dw_^−1^) generally revealed lower values, those of Serbian^55^ (LOQ – 670 μg kg_dw_^−1^ in 2013; LOQ – 115 μg kg_dw_^−1^ in 2023; soil depth, 0–30 cm) monitoring campaigns were in the same range as in ours. Frequencies of occurrence varied as well, probably due to different LOQs of the applied analytical methods. The TP trifloxystrobin CGA was detected in 34% of the samples from 1.1 to 95 μg kg_dw_^−1^ (median: 4.5 μg kg_dw_^−1^). Sampling time s1 showed significantly lower concentrations in comparison tos3. This high detection frequency of trifloxystrobin's TP is obvious due to its moderate persistency (DT_50,field_: 70 days), while the parent AI has a very short half-life of 1.7 days (Table S3). In summary, the occurrence and temporal variability of soil residual concentrations of these prevailing individual AIs over several potato CPs largely reflect the corresponding applications for plant protection, which is now systematically elaborated in the next chapter.

### Application–detection scenarios

From the 43 AIs applied (chapter S2 and Fig. S5), 18 were F, 12 were H, and 13 were I. Eleven F, four H and one I coincided with the list of 31 AIs (excl. TPs) analysed. Hence, residues could be measured for more than half of the applied AI, which was similar to other studies on pesticide use under potato production.^56^

The analysis of 31 AIs in a total of 38 sites (CP1–3) resulted in 1178 cases to evaluate. Detailed information about application amounts of AIs during CP1–3 (data of CP4 not available), including a comparison with FAO data and research articles of applications in tropical and temperate climate, can be found in the SI, chapter S2.

True-negatives (not applied-not detected, white cells in Fig. 1) were predominant (72% or 854 cases). Six compounds, benthiavalicarb-isopropyl, fenamidone, clomazone, deltamethrin, pirimicarb, and spirotetramat, were analysed, but neither were applied nor detected. Similar proportions (71%^18^ and 68%^38^) of true-negative cases were obtained in archived agricultural soils of Switzerland.

True-positives (applied-detected, colored cells with a grey circle; Fig. 1) constituted 11% (126 cases). Generally, most true-positives were found in CP3 for azoxystrobin, benalaxyl, chlorothalonil, and *S*-metolachlor, while CP2 showed the most for ametryn and CP1 for cyproconazole. *S*-Metolachlor had the highest number of true-positives (53%, Fig. S6), followed by azoxystrobin (50%), cyproconazole (37%), chlorothalonil (37%), and ametryn (32%). Cyproconazole was detected in soil samples whenever it was applied, only showing true-positives and true-negatives (Fig. 1, S6). For these five AIs (*i.e.*, *S*-metolachlor, azoxystrobin, cyproconazole, chlorothalonil, and ametryn), half-lives were calculated (next chapter).

False-positives (not applied-detected, colored cells without a grey point; Fig. 1) were found in 12% (140 cases). The far most false-positives were counted for dicofol (30 cases, Fig. S6). According to the farmers' records, this pesticide was last applied around 10 years ago, and although it is only moderately persistent (DT_50_ 80 days, which is not derived from the field, Table S3), it still appeared in soil samples at concentrations between 0.14 and 35 μg kg_dw_^−1^. This is in line with the detection of dicofol of up to 50 μg kg_dw_^−1^ in Chinese mountainous forest soils in climate types ranging from humid continental to humid subtropical and semiarid continental,^54^ where it was never applied, indicating long range transport and ubiquitous background concentrations. The behaviour resembled one of its intermediates during production, dichloro-diphenyl-trichloroethane, another organochlorine I. However, DT_50_ obtained from lab or field dissipation studies over limited periods of time (usually around 120 days) may fall short of predicting long-term residues of many pesticides, as was shown for atrazine, *S*-metolachlor, and others in temperate soils.^38^ False-positives were even more surprising in this study as remains of AIs in tropical soils might show shorter DT_50_ than indicated in the PPDB^44^ with field soils of temperate climate (see below). Other reasons for false-positives (Fig. S6) could be cross contamination, for example, from spray drift^57^ (S19 exposed to wind (no aerial pesticide applications in Cuba) of sites S17 and S18 (Fig. S1), where, for instance, azoxystrobin or chlorothalonil was applied (Fig. 1)), an application of the same AI to a preceding crop on the same field (Fig. S2), and/or farmers could have missed to record the application.

False-negative (applied-not detected, empty cells with a grey point; Fig. 1) were 58 cases (5%) and bifenthrin accounted for the highest number (16 cases, Fig. 1, S6). It had only four true-positives at low concentrations (7–12 μg kg_dw_^−1^), which was unexpected because a DT_50,field_ of 87 days (Table S3) and a sampling close to the application time should result in 100% true-positives. However, according to eqn (1), *A*_0_ of bifenthrin was low (median, 0.01 kg_AI_ ha^−1^; chapter S2) because of (i) low application rates, (ii) a high *f*_int_ (>0.5) around 50 days after planting potato (FOCUS workgroup^45^), and/or (iii) residual soil concentrations below the LOQ (5 μg kg^−1^).^28^

Seven TPs were analyzed with its respective AI applied at 38 sites, which resulted in 266 cases. Similar to AIs, true-negatives were also predominantly counted for TPs (78%, 207 cases), followed by false-positives with 9% (24 cases). True-positives accounted for 8.6% (23 cases), which were trifloxystrobin and metribuzin, mostly detected in CP1. Trifloxystrobin CGA was detected whenever trifloxystrobin was applied (14 true-positive cases) due to TP's moderate persistency (see above), and the seven false-positives (Fig. S6) were probably due to earlier applications of trifloxystrobin for which the residue fell below the LOQ. The single true-positive of 2,6-dichlorobenzamide corresponded to the single true-positive of fluopicolide. The behaviour of TPs of metribuzin was mainly the same as for their parent compound. However, metribuzin DA had only one true-positive case and nine false-negatives due to the high LOQ (25 μg kg^−1^).^28^ For the other three TPs included in our method (Fig. 1), no reports of parent AI applications existed, neither were they ever detected.

### Observed half-lives of frequently applied and detected pesticides in Cuban soils

**Fig. 2:**
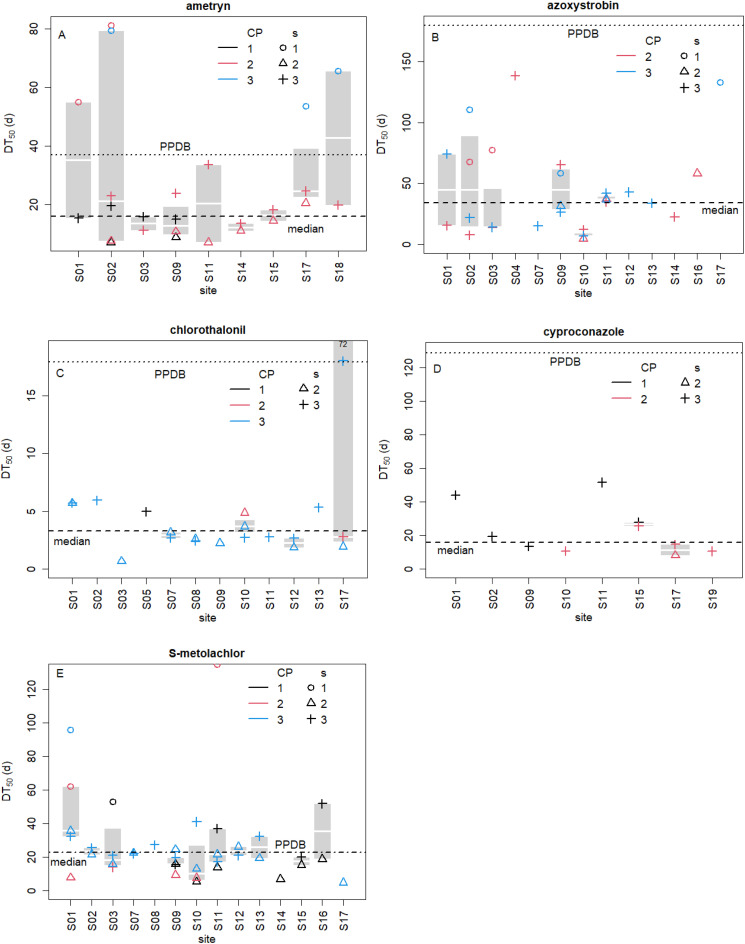
Half-lives (DT_50_, *d*, panels A–E) of the pesticides most frequently detected in soils under potato cultivation, *i.e.*, ametryn (herbicide (H), *n* = 25), azoxystrobin (fungicide (F), *n* = 27), chlorothalonil (F, *n* = 20), cyproconazole (F, *n* = 10), and *S*-metolachlor (H, *n* = 38) observed at different sites. Only individual true-positive cases (applied-detected) were used for the boxplots. Data points are categorised according to three sampling times: before planting potato (s1, circle), during peak pesticide application (s2, triangle), around harvest (s3, cross) and cultivation period: 2018–2019 (CP1, black), 2019–2020 (CP2, red), and 2020–2021 (CP3, blue). If a sampling time was not depicted, the residue fell below the limit of quantification and/or was not a true-positive case. The boxes represent the 25^th^ to 75^th^ percentile, and the bold white line dividing the box is the median of single data points. Geometric means of data from single sites served to indicate the median over all sites (dashed line). Dotted line shows DT_50,field_ of the Pesticide Properties Database (PPDB^44^; for *S*-metolachlor, the line of geometric mean and the PPDB^44^ coincide).

The degradation rates (*k*, 1 d^−1^) of ametryn and S-metolachlor were fitted according to eqn (2) and for azoxystrobin, chlorothalonil, and cyproconazole according to eqn (3a)–(3d). Observed half-lives (DT_50,obs_) were calculated according to eqn (4) and shown in boxplots in Fig. 2 for individual sites categorized by CP and sampling time. The DT_50,obs_ of ametryn ranged from seven to 81 days (Table S8; DT_50,median_: 16 days), azoxystrobin from five to 138 days (DT_50,median_: 34 days), chlorothalonil from one to 72 days (DT_50,median_: 3.3 days), cyproconazole from eight to 52 days (DT_50,median_: 16 days), and *S*-metolachlor from five to 135 days (DT_50,median_: 23 days). While the DT_50,median_ of *S*-metolachlor coincided with the value from the PPDB^44^ (dashed-pointed line), the other four AIs had up to eight times (cyproconazole) lower values than the DT_50,field_. Observed half-lives were smaller when the time between application and sampling was lower, which is well visible for ametryn, azoxystrobin, and *S*-metolachlor. Ametryn and *S*-metolachlor, pre-emergence H, were applied before s2, and residues at s3 (crosses) or s1 (circles) were results of “pseudo-applications”. Almost a complete “series” of DT_50,obs_ over three campaigns were visible for ametryn at site S02. The DT_50,obs_ values shown in s2 and s3 of CP1 (black triangle and cross) were lower that at s1 of CP2 (red circle). Site 02 experienced another application of ametryn in CP2, which resulted in lower DT_50,obs_ (red triangle) at s2 than at s3 (red cross) and at s1 in CP3 (blue circle). In CP3, ametryn was not applied (Fig. 1), as indicated by the absence of blue triangles and crosses. Typically, for long periods of around 200 days from s3 of the former to s1 of the following CP, the DT_50,obs_ values were markedly higher (up to 11 times, *S*-metolachlor, S11) than the DT_50,median_ or DT_50,field_ of the PPDB.^44^ Residues reflecting such high DT_50_ were notably higher than a few percent ranging up to 30% after application, as was also found in Riedo *et al.*^38^ and Mangold *et al.*^40^ This behaviour points towards a biphasic degradation, as reported in Laabs *et al.*^58^ In summary, s1 seemed to be the main reason for the variation of DT_50,obs_. By removing s1 values of the H and azoxystrobin and outliers of the latter and chlorothalonil, DT_50_ decreased a quarter (ametryn) to a third (azoxystrobin, geometric mean over all sites, Table S8). These DT_50,median_ values were conservatively evaluated and represented robust values, because firstly only true-positives (11% of scenarios) were taken into account and not false-negatives (applied but not detected, 5%), which could have decreased the DT_50_. Secondly, the sensitivity analysis of the *A*_0_, where soil depths and *f*_int_ were varied (Table S9), showed remarkable stability of the DT_50,median_ with values remaining far below the DT_50,field_ of the PPDB.^44^ This was mainly due to the many pseudo-applications.

Hence, persistent compounds in temperate climate such as, for instance, azoxystrobin and cyproconazole might still degrade in reasonable time periods in the tropics. Lower dissipation rates in temperate than in tropical climate were reported before^2,26,58^ and compared well with those of this study. Ametryn in a banana plantation in St. Lucia, the Caribbean, was three to eight days.^59^ The DT_50_ of azoxystrobin was reported for 16 days^60^ or nine to 13 days^61^ in tropical soils of China where banana was grown. Chlorothalonil's DT_50_ was 2.2–3.9 days in Costa Rican banana plantations.^62^ The DT_50_ values of cyproconazole in an artificial soil without stereoselectivity and diastereoselectivity were around eight days^63^ at 600 μg kg^−1^ application and 20–24 days at 6000 μg kg^−1^. *S*-Metolachlor's DT_50_ values in sugarcane fields of Florida, USA, were 12 to 24 days in mineral soils and 50 to 126 days in organic soils.^64^ In temperate climate (Switzerland), azoxystrobin's half-life was recently found to be 41 days,^40^ which was closer to this study than the value from the tropics. Although comparison of our DT_50,obs_ with the literature was difficult due to the lack of matching conditions such as AI, field trials in test plots,^60,61^ lab experiment,^63^ other crops than potato^60–62^ or sampling times only for one CP^40,60–63^, the half-lives were comparable.

### Influential variables potentially explaining pesticide occurrence

In essence, results from the above chapters could mainly be explained by pesticide application regimes and physico-chemical properties of the compounds. This is in line with findings of, for instance, Materu *et al.*^5^ for Tanzania and even Hvězdová *et al.*^19^ who worked in temperate climate. Biplots of the PCA showed the substances in concert with the important factor(s) identified (Fig. S7A) and optimized (Fig. S7B). While trifloxystrobin CGA, dicofol and oxyfluorfen in Fig. S7B rather discriminate along the solubility and *K*_fOC_ axis (horizontal axis), azoxystrobin, cyproconazole and *S*-metolachlor discriminate along their DT_50,PPDB_^44^ (vertical axis) where the former two have high half-lives and the latter rather shows low half-lives in comparison with the majority of the detected pesticides. Vapour pressure and the sum of applications are less correlated with the other three factors. Azoxystrobin, dicofol, dimetomorph, *S*-metolachlor, and trifloxystrobin CGA were mostly detected in all four campaigns. These compounds had either high *K*_fOC_, DT_50,PPDB_, solubility, or vapour pressure or were applied in high amounts. Although *S*-metolachlor and trifloxystrobin CGA have high solubility and vapor pressure, facilitating dissipation, they were nevertheless frequently detected. Application amounts and a moderate persistency, respectively, might have overruled these properties (Table S3). Furthermore, the most true-positive cases were counted for ametryn, chlorothalonil, *S*-metolachlor, azoxystrobin cyproconazole, and benalaxyl. The first three showed high application amounts, while azoxystrobin had a high DT_50,PPDB_, and the latter two had no specific properties in comparison to the others (place towards the origin of the biplot). The high *K*_f,OC_ (Table S3) of ametryn additionally contributed to this result as well as the one of dicofol, having the most false-positives. It remained detectable even 10 years after non-use. It is plausible to assume that ametryn and dicofol sorbed strongly to OC due to their high *K*_f,OC_, resulting in limited availability for degradation.

Furthermore, DT_50,obs_ values were opposed to soil properties such as OC, pH, texture and BR and MB. Data scattered randomly (Fig. S8–S12) and in contrast to Laabs *et al.*,^58^ who also worked under tropical conditions in Brazil, no positive correlation could be found with, *e.g.*, half-lives and the clay content. However, the researchers worked with a soil with low to intermediate clay contents, while soils in this study had a much broader range of 10–82%. The fact that no trend can be derived from the above findings underlines the statement of Racke *et al.*^2^ that tropical soils defy easy generalisations concerning the fate of pesticides.

### Risk quotients of compounds to earthworms (*E. fetida*)

**Fig. 3:**
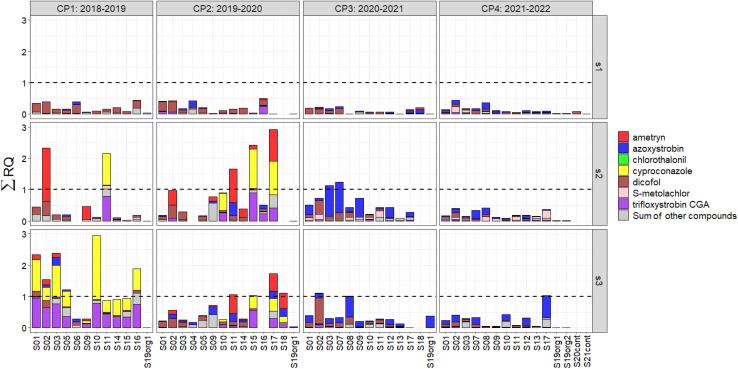
Chronic risks for earthworms (*Eisenia fetida*) expressed as sum of risk quotients (*Σ*RQ according to eqn (7)) in soils of 18 conventional potato production sites (S01 to S18), where “org” means organic potato production and “cont” means the control site, at three sampling times: before planting potato (s1), during peak pesticide application (s2) and around harvest (s3) over four consecutive cultivation periods (CP1–CP4). Colours indicate the most important compounds concerning the RQ as well as the *Σ*RQ of the rest of the substances analysed in this study (main text and Table S10). Dashed line indicates *Σ*RQ = 1, above which a potential risk is posed.

The soils analyzed, CP1–4 and s1–s3, showed a potential ecological chronic risk for earthworms mostly at s2 and s3 in CP1 and CP2 (Fig. 3 and Table S10). The risk emanating from pesticide residues in soil was classified as follows: *Σ*RQ (eqn (7)) <0.01 indicated negligible, 0.01 < *Σ*RQ < 0.1 low, 0.1 < *Σ*RQ < 1 medium, and *Σ*RQ > 1 high ecological terrestrial risks.^65^ In s1, none of the sites exhibited a high risk, as well as in s2 and s3 of CP4. However, for CP1–3, seven sites in s2 and 13 in s3 had a high risk. This finding was expected as pesticide residues were higher before s2 and s3 than before s1 (Fig. S2–3). The compounds that mostly contributed to the *Σ*RQ were ametryn and cyproconazole in s2 of CP1 and CP2, cyproconazole in s3 of CP1, and azoxystrobin in s2 and s3 of CP3. Again, dicofol was prominent in RQs of soils mostly in s1 as background contamination. Not only false-positives but also false-negatives could pose a problem and lead to RQ > 1 for short periods as in the case of trifloxystrobin (short-lived AI applied but not detected, Fig. 1) degrading into trifloxystrobin CGA predominating s3 in CP1. It is difficult to compare our RQs directly with literature data, but the estimated risks based on predicted environmental concentrations emanating from up to 124 pesticides applied in Cuba to crops, such as sugarcane, rice, vegetables, cereals, tubers and root vegetables, and fruits from 2011 to 2014 to earthworms were considered relatively low (0.01–1.0) in comparison to aquatic organisms or bees.^66^ In conclusion, the risk posed by pesticides to soil organisms is highly dependent on the cocktail applied and dominated by relatively few key compounds (*i.e.*, ametryn, azoxystrobin, cyproconazole and dicofol) thoroughly investigated in this study. Ametryn was also reported to be among those posing risks in a Cuban ecotoxicity study.^66^ Moreover, and as demonstrated with sampling times s1–3, risk should not be perceived as static and constant but is subject to pronounced seasonal changes^67^ and environmental conditions.

## Conclusions

This systematic study under real environmental conditions added evidence for higher dissipation rates in tropical than in temperate soils and AIs such as azoxystrobin and cyproconazole turning from persistent to moderate to non-persistent in the tropics. Despite the reduced DT_50,obs_ in comparison to temperate climate, RQs > 1 were present in s2–3. Moreover, the risk assessment based on effect data of temperate climate might not be adequate for tropical soils.^26^ Additionally and according to Pelosi *et al.*,^49^ the RQs were underestimated^68^ because there are other soil dwelling species more sensitive than *E. fetida* to pesticides. This is all the more important as soil pollution, pesticides and metals, posing the largest negative effect on soil biodiversity, accounting for about 60% of global biodiversity.^69^

These findings suggest to initiate (soil) monitoring database(s) of pesticides for tropical climate, adapt, and include DT_50_ of AI in tropical climate to databases such as the PPDB.^44^ Additionally, standard pesticide risk assessment for tropical soils could be adjusted or even loosened. Campan *et al.*^70^ recently stated to adjust the Arrhenius equation for lower activation energy *E*_a_ in degradation models for the tropics where temperatures >20 °C in soil are normal.^70^ This is even more important in the future, as climate change influences not only the necessity for pesticide application but also its dissipation kinetics.^71^

Pesticides should be monitored in cooperation with farmers who directly provide application data to cooperatives to make them transparent, as official sources as, for instance, the FAO may substantially underestimate the pesticide use, especially in low income countries, due to a recent restructuring of the agrichemical supply chain and agrarian development.^72^ Moreover, data should be of high quality from application over a sampling design adapted to the purpose (background concentration monitoring *versus* peak application time) for environmental risk assessments in soils and results communicated to farmers, legislators, and environmental agencies. After all, there is increasing demand for *ex ante* impact pathways that are place-based, coherent, and plausible to foster effective interventions.^73^ A potential forming of a Central and South American pesticide network^32^ can help understand the pathway from application to detection of pesticides and their impact on the environment. This shall lead to reasonable RGVs, thus not dispersing over several orders of magnitude.^25^ Such a network should also raise awareness among farmers on the use, risk, and hazards associated with human exposure to pesticides,^74^ to improve ONE HEALTH.

## Author contributions

Brizeidi Peña: conceptualization, data curation, formal analysis, funding acquisition, investigation, project administration, resources, validation, visualization, writing – original draft; Isabel Hilber: conceptualization, formal analysis, funding acquisition, methodology, project administration, resources, software, validation, visualization, assisted in writing – original draft, writing – review & editing; Dayana Sosa: conceptualization, formal analysis, funding acquisition, investigation, methodology, project administration, resources, validation, visualization, assisted in writing – original draft, writing – review & editing; Arturo C. Escobar: conceptualization, funding acquisition, methodology, project administration, resources, writing – review & editing; Thomas D. Bucheli: conceptualization, funding acquisition, methodology, project administration, resources, supervision, visualization, writing – review & editing.

## Conflicts of interest

The authors declare no conflicts of interest.

## Supplementary Material

EM-027-D5EM00119F-s001

EM-027-D5EM00119F-s002

## Data Availability

The data supporting this article have been included as part of the SI. Supplementary information is available: applied and quantified AI (TP) for each site, cultivation period (CP), and sampling time point (d5em00119f1.xlsx); further details on analysis of physico-chemical and biological soil properties (Text S1), applied pesticides to potato crops in Mayabeque, Cuba (Text S2), sampled farm sites (Table S1), soil parameters (Table S2), chemical-physical properties of analyzed pesticides (Table S3), nlme model results (Tables S4 & S5), effect data for earthworms (Table S6), pesticides applied to preceding crops (Table S7), summarized observed half-lives (Table S8), sensitivity analysis of observed half-life calculations (Table S9), chronic risk of earthworms (Table S10), map with site locations in investigated counties (Fig. S1), concentrations of pesticides summed up by type (Fig. S2), boxplots of concentrations of all analytes grouped by CP, sampling time point and pesticide type (Fig. S3), similar boxplots for the most frequently detected compounds (Fig. S4), sum of dosages of applied AI per site and CP (Fig. S5), relation between AI applied and detected (Fig. S6), principal component analysis of compounds analyzed (Fig. S7), correlations of soil properties and half-lives of the most frequently detected compounds (Fig. S8–12) (d5em00119f2.pdf). See DOI: https://doi.org/10.1039/d5em00119f.
