# Supplementary material for: Extended pesticide soil monitoring in Cuban potato (Solanum tuberosum L.) production: residue co-occurrence, dissipation rates, ecological risks, and implications
Source: Environ Sci Process Impacts. 2025 Aug 13;27(9):2685–97. doi: 10.1039/d5em00119f (PMC12371700; doi:10.1039/d5em00119f)
Supplement: EM-027-D5EM00119F-s002 [file EM-027-D5EM00119F-s002.pdf]

Supplementary Information (SI) for Environmental Science: Processes & Impacts.
This journal is © The Royal Society of Chemistry 2025

Supporting Information

Extended pesticide soil monitoring in Cuban potato (*Solanum tuberosum* L.) production: residue co-occurrence, dissipation rates, ecological risks, and implications

Brizeidi Peña[1], Isabel Hilber[2], Dayana Sosa[1]*, Arturo C. Escobar[1] and Thomas D. Bucheli[2]*

Affiliation:
1 Centro Nacional de Sanidad Agropecuaria (CENSA), Unidad Analítica de Residuos y Contaminantes Apartado 10 CP 32700 San José de las Lajas, Mayabeque Cuba
2 Agroscope Environmental Analytics, Reckenholzstrasse 191, 8046 Zurich, Switzerland

Corresponding author:
Thomas D. Bucheli, Environmental Analytics, Agroscope, Zurich, Switzerland; orcid.org/0000-0001-9971-3104; Phone: +41 58 468 7342; Email: thomas.bucheli@agroscope.admin.ch

Dayana Sosa Pacheco, Unidad Analítica de Residuos y Contaminantes CENSA, San José de las Lajas, Mayabeque; orcid.org/0000-0002-8867-0492; Email: dayanasosap@gmail.com

S1. Analysis of physico-chemical and biological soil properties

Soil physico–chemical properties such as bulk density (bd), organic carbon content (OC), pH, and texture were analyzed according to the reference methods of Agroscope. (1) The bd was measured by the volume and dry weight of the fine soil. Organic carbon was determined by the modified Walkley-Black method. The pipetting method served to determine clay (<2 µm), silt (2 to 50 µm) and sand (>50 µm). Soil pH was determined in a Metrohm robotic titrosampler (Herisau, Switzerland).

Biological soil properties like basal respiration (BR) and microbial biomass (MB) of fresh soils at 60% water holding capacity were analyzed by the Cuban team at CENSA laboratories. The RB was evaluated by pre–incubating the soil samples for seven days. After that, soils were incubated in a closed system with a NaOH solution for seven days. The addition of $BaCl_2$ produced the precipitation of Na_2CO_3, and the NaCl produced in the reaction was titrated with HCl and the results were calculated as equivalent to CO_2 produced by microorganisms.(2) The MB was carried out with the chloroform–fumigation-extraction method for microbial cell destruction during seven days according to Vance et al.(3) Potassium sulphate was used for cell extraction, equivalent to microbial carbon content. Fumigated and no-fumigated samples were compared to determine the content of extracted OC by spectrometry UV-VIS at 600 nm.

S2. Applied pesticides to potato crops in Mayabeque, Cuba

In total, 43 AIs were applied to sites conventionally managed during the three cultivation periods (CP, Figure S5). Between two and 17 AIs were used **at a site per CP** (median 11) in sums ranging from 0.001 to 26 kg_{AI}/ha (median 0.2 kg_{AI}/ha). This range was broader than reported for earlier years (2011 to 2013) and the median lower.(4) These application concentrations to potato crops in Cuba were comparable to others such as Canada,(5)Ecuador,(6) and Costa Rica.(7) The Food and Agricultural Organisation (FAO) indicated on its website an estimated value for Cuba of 0.75 $kg_{AI}/ha_{cropland}$(8) from 2018 to 2022, each. This is in the same order of magnitude as the median in this study, although the crop is not indicated in the FAO database. The FAO estimated a narrower range than the one in this study but shifted towards the upper limit for the total use of pesticides in Costa Rica with 17 and 23 kg_{AI}/ha and of Columbia between 8.2 and 16 kg_{AI}/ha between 2018 and 2022. Although these values were all estimated by the FAO and the crop species was not indicated either, the application concentrations for countries in the Caribbean were comparable.

Eighteen fungicides (F), 12 herbicides (H) and 13 insecticides (I) were applied (Figure S5), 15 F, nine H and seven I analysed (Figure 1), and 11 F, four H and one I of the latter applied (Figure 1, grey circles). This order was similar in other studies where potatoes were produced.(9) The sum of F per site and CP ranged from 0.4 to 11 kg_{AI}/ha with a median 1.7 kg_{AI}/ha. Herbicides presented a range of 0.2 to 3.9 kg_{AI}/ha and a median 1.6 kg_{AI}/ha. Bifentrin (I) concentrations oscillated between 0.01 and 0.09 kg_{AI}/ha with a median of 0.01 kg_{AI}/ha. Eight AI were applied at >30% per CP and site (Figure 1), which were azoxystrobin (F, applied in 63% of conventional sites (min, max, median in kg_{AI}/ha: 0.06, 1.2, 0.3), S-metolachlor (H, 60%, 0.2, 1.9, 0.96), bifenthrin (57%, 0.01, 0.09, 0.01), tebuconazole (F, 45%, .0.02, 0.5, 0.1), chlorothalonil (F, 45%, 0.8, 10.0, 3.4), cyproconazole (F, 40%, 0.02, 0.7, 0.2), trifloxystrobin (F, 40%, 0.05, 1.7, 0.5), and ametryn (H, 34%, 1.6, 2.4, 2.0). These amounts were common when compared to the literature. Cyproconazole was applied at 0.1 kg_{AI}/ha for tuber crops in a study of the European Food Safety Autority (EFSA).(10) The application authorization for this AI expired in the European Union May 2021. In contrast, in a study from Costa Rica, a mean of 0.0005 kg_{AI}/ha, 6.4 kg_{AI}/ha and 0.003 kg_{AI}/ha of azoxystrobin, chlorothalonil and cyproconazole, respectively were applied to potato crop during the period from 2006 to 2009.(7) While the Cuban application concentration

medians of azoxystrobin and cyproconazole where magnitudes higher than in Costa Rica, the one of chlorothalonil was in the same range.

Mancozeb, imidacloprid, abamectin, and propaquizafop were compounds most frequently applied (86%, 63%, 54%, 51%; Figure S5). These four AI were not analysed because they were either not compatible with GC-MS/MS (i.e., abamectin, mancozeb, and imidacloprid) and/or degraded before analysis due to a very short half–life (i.e., mancozeb ($DT_{50\ lab}$ 0.015–0.74 days(11)) and propaquizafop ($DT_{50\ lab}$ 0.09 to <3 days(11))).

Table S1: Sampled farm sites (S01 to S21), their county (Batabanó (BT), Quivicán (QV), and San José de las Lajas (SJ), Mayabeque, Cuba), latitude, longitude, participation in the cultivation period (CP1–4), and a general description of the site. While S01 to S18 were conventionally managed, S19org was under organic production, and S20cont and S21cont were forest sites serving as control as they had no agricultural influence. Cultivation periods were from 2018–2019 (CP1), 2019–2020 (CP2), 2020–2021 (CP3) and 2021–2022 (CP4).

Site	County	Lat (º)	Lon (º)	Cultivation period	General characteristics of the site
**S01**	BT	22.75345	-82.27111	CP1-4	In the surroundings were little vegetation, groves in the distance and potato and beans near the sampling site. Corn and sweet potato were rotation crops
**S02**	BT	22.77891	-82.25921		
**S03**	BT	22.793371	-82.26248		
**S04**	BT	22.73176	-82.3547	CP2	Presence of the different crops in the surrounding as carrot, onion, beet, abundant vegetation with permanent trees. Rotation crops were cucumber, potato and banana.
**S05**	BT	22.74835	-82.25058	CP1-2	Abundant vegetation in the farm. Presence of permanent crops of fruit trees as avocado, coconut, pear, apple, guava (big field), mango, papaya, banana, corn and timber trees. Rotation was with corn, tomato, potato and papaya.
**S06**	BT	22.72958	-82.256	CP1-2	Around the farm, other potato fields of other farms were present. Also, there were banana in the distance. Corn was the crop rotation.
**S07**	BT	22.797518	-82.241127	CP3-4	In the surroundings there were little vegetation, groves in the distances and a presence of crops near of sampled site as potato. Corn was the rotation crop.
**S08**	BT	22.773056	-82.319427	CP3-4	In the surroundings there were a lot of trees, and also other farms near with potato planted. Corn and beans were the rotation crops.
**S09**	QV	22.85835	-82.39561	CP1-4	Presence the other fields with potato but also with corn, beans and chickpea. Far away permanent crops. Corn was the rotation crop.
**S10**	QV	22.87529	-82.39731		Presence the trees in the surroundings and the others crops as banana, casaba, tomato, beans and potato. Corn was the rotation crop.
**S11**	QV	22.81494	-82.456283		Potato was also planting in the surroundings farms and banana was on front. Corn and sweet potato

					were the rotation crops.
**S12**	QV	22.48128	-82.2665	CP3-4	Potato was also planting in the surroundings farms. Groves in the distances and a little presence of crops as banana.
**S13**	QV	22.837044	-82.388382	CP3-4	Presence of banana in the surroundings, also there were other field with sweet potato, cucurbit. Beans was the rotation crop.
**S14**	QV	22.82806	-82.38035	CP1-2	Abundant vegetation, presence of banana, guava, cherry, eggplant, tomato, cucumber (sometimes), carrot, and others permanent. Corn, sweet potato and carrot were the rotation crops.
**S15**	QV	22.84679	-82.38959	CP1-2	Presence of vegetation in the surroundings, other field near with sweet potato, beans and banana. Rotation with casaba, sweet potato and corn.
**S16**	QV	22.76337	-82.3641	CP1-2	Few vegetation, in the distance groves. Banana, cabbage present in the surroundings. Rotation with beans, tomato and sweet potato.
**S17**	SJ	22.98551	-82.13493	CP2-4	Presence of the other field with potato in the surrounding. Presence of the different crops in the surrounding as sweet potato, banana, cassava, guava, and lemon. Rotation with beans and corn.
**S18**	SJ	22.98854	-82.13317	CP2-3	The site belongs to the same farm as S17 and is near S17. Presence of the other field with potato in the surrounding. Presence of the different crops in the surrounding as sweet potato, banana, cassava, guava, and lemon. Rotation with corn.
**S19org**	SJ	23.011172	-82.1392	CP1-4	Abundant vegetation and land rotation, presence of mango, guava, avocado, coffee, banana, beans, cabbage, pepper, corn, groves, and palms.
**S20cont**	BT	22.751726	-82.276695	CP4	Forest site near S01
**S21cont**	SJ	22.989508	-82.151108		Forest site at the CENSA institution

Table S2: Soil sampling at sites S01–S18 were conventionally and organically (S19org1) managed. Soil was sampled at cultivation period (CP)1 2018–2019, CP2 2019–2020, and CP3 2020–2021 at indicated dates. (Please note that application data were not available for CP4.) Sampling times were before planting potato (s1), peak pesticide application (s2) and harvest (s3). Determined properties of soils were bulk density (bd), organic carbon (OC) content, pH, sand, silt, clay and microbiological properties such as microbial biomass (MB) and basal respiration (BR). Observed half-lives ($DT_{50,obs}$) of the five most important active ingredients were calculated acc. to eq. 4 (main text). Empty cells indicate no results available.

Site	CP	s	Sampling date	bd	OC	pH	Sand	Silt	Clay	BR	MB	Ame-tryn	Azoxy-strobin	Chloro-thalonil	Cypro-conzaole	S-metola-chlor
				[g/cm^3]	[%]	[-]	[%]			[mg_{CO2}/kg_{soil} *day]	[mg_C/kg_{soil}]	$DT_{50,obs}$ [d]				
S01	1	1	26-10-2018	0.82	1.53	7.82	15.20	39.60	42.60		198					
		2	14-12-2018	0.82	1.12	7.59	11.70	51.40	35.00	19	410					
		3	14-3-2019	0.82	1.18	7.87	13.80	52.20	32.00	69	653	15.35			44.09	
	2	1	19-9-2019	0.82	1.66	7.74	9.90	18.00	69.20	65	404	54.93				61.34
		2	21-1-2020	0.82	1.65	7.66	13.20	22.20	61.80	13	91					7.85
		3	26-2-2020	0.82	1.40	7.73	10.10	25.10	62.40	88	587		15.68			
	3	1	9-9-2020	0.82	1.29	7.73	12.90	20.20	64.70	56	596					96.08
		2	13-1-2021	0.82	1.69	7.71	10.70	20.90	65.50	13	507			5.70		35.82
		3	23-2-2021	0.82	1.63	7.82	6.10	11.40	79.70	37	670		74.11	5.73		32.54
S02	1	1	1-11-2018	0.86	1.47	7.81	9.20	31.00	57.30	30	414					
		2	8-1-2019	0.86	1.29	7.72	13.00	46.20	38.60	796	924	7.03				
		3	14-3-2019	0.86	1.35	8.11	13.30	52.90	31.50	32	376	19.50			19.41	
	2	1	17-10-2019	0.86	1.62	7.69	10.50	7.20	79.50	22	461	81.28	67.63			
		2	20-1-2020	0.86	1.61	7.92	10.90	13.70	72.60	63	537	7.54				
		3	3-3-2020	0.86	1.26	7.81	11.50	8.90	77.40	44	53	22.87	7.79			
	3	1	16-9-2020	0.86	1.65	7.53	10.50	11.70	75.00	25	601	79.38	110.42			
		2	13-1-2021	0.86	1.83	7.39	12.30	23.90	60.60	38	537					21.70

		3	24-3-2021	0.86	2.37	7.49	9.90	20.80	65.20	69	808		21.96	5.95		25.68
S03	1	1	2-11-2018	0.94	1.24	7.67	14.80	37.00	46.10	18	170					53.24
		2	18-12-2018	0.94	1.18	7.41	12.80	46.50	38.70	54	471					
		3	15-3-2019	0.94	1.00	7.67	21.10	56.20	21.00	25	453	15.79				
	2	1	26-9-2019	0.94	1.48	7.70	17.60	42.70	37.10	63	484		77.21			
		2	29-1-2020	0.94	1.68	7.78	7.10	14.30	75.70	82	554					
		3	17-3-2020	0.94	1.66	7.72	18.10	36.60	42.40	13	955	11.18	13.94			14.19
	3	1	9-9-2020	0.94	1.47	7.65	18.70	39.60	39.20	35	335					
		2	17-2-2021	0.94	1.78	7.78	16.70	37.10	43.10	54	320			0.68		15.95
		3	5-4-2021	0.94	2.50	7.81	11.40	27.80	56.50	32	320		14.37			21.09
S04	2	1	27-9-2019	0.84	1.82	7.64	21.60	59.10	16.20	11	500					
		3	16-3-2020	0.84	1.61	7.79	24.20	52.40	20.60	44	525		138.19			
S05	1	1	8-11-2018	0.87	1.24	7.81	12.30	23.70	61.90	16	200					
		2	22-11-2018	0.87	1.24	7.59	11.80	28.60	57.50	28	16					
		3	7-2-2019	0.87	1.29	7.86	16.10	31.40	50.30	19	639			4.99		
	2	1	6-9-2019	0.87	1.24	7.67	18.90	15.00	64.00	32	293					
		2	15-1-2020	0.87	1.46	7.53	17.50	14.50	65.50	75	463					
		3	10-3-2020	0.87	1.33	7.61	19.40	14.70	63.60	88	394					
S06	1	1	9-11-2018	0.84	1.41	7.24	9.10	64.70	23.80	12	102					
		3	12-2-2019	0.84	1.06	7.54	13.70	60.20	24.30	88	505					
S07	3	1	16-9-2020	0.77	1.87	8.05	12.10	39.50	45.20	19	553					
		2	2-10-2021	0.77	1.60	8.14	14.40	45.10	37.80	57	537			3.18		22.53
		3	20-3-2021	0.77	1.47	8.13	8.70	31.30	57.50	25	874		15.27	2.70		22.29
S08	3	1	24-9-2020	0.92	1.77	7.71	15.50	54.40	27.10	25	549					
		2	2-10-2021	0.92	1.76	7.65	18.60	50.80	27.60	88	595			2.61		
		3	6-4-2021	0.92	2.23	7.83	7.40	26.90	61.90	75	367			2.46		27.47
S09	1	1	26-11-2018	0.83	1.06	7.59	15.50	57.00	25.70	3	42					

		2	25-1-2019	0.83	1.12	7.64	16.50	27.90	53.70	57	665					15.97
		3	11-3-2019	0.83	0.94	7.66	16.30	57.30	24.80	60	521				13.58	15.76
		1	20-11-2019	0.84	1.48	7.57	28.50	49.50	19.50	76	509					
	2	2	19-2-2020	0.84	1.43	7.47	24.70	56.30	16.50	13	565	8.75				9.48
		3	24-3-2020	0.84	1.40	7.35	27.70	55.30	14.60	13	457	14.93	65.49			
		1	10-9-2020	0.83	1.40	7.36	19.70	55.80	22.10	88	615		58.60			
	3	2	2-3-2021	0.83	1.55	7.35	20.70	51.20	25.40	75	572	10.70	31.61	2.25		24.71
		3	25-3-2021	0.83	2.31	7.63	11.20	58.50	26.30	50	371	23.84	26.52			19.81
		1	28-11-2018	0.81	1.12	6.24	17.30	43.80	37.00	15	188					
	1	2	21-1-2019	0.81	1.06	6.12	14.00	41.40	42.80	74	789					5.39
		3	18-3-2019	0.81	1.12	6.13	19.10	48.90	30.10	43	431					
		1	20-11-2019	0.83	1.25	6.17	35.30	43.00	19.60	11	415					
S10	2	2	19-2-2020	0.83	1.33	6.06	30.50	36.60	30.60	13	499			4.85		7.64
		3	18-3-2020	0.83	1.08	6.10	29.70	37.20	31.20	101	451		12.25		10.80	
		1	30-9-2020	0.81	1.24	6.37	26.60	43.70	27.60	38	635					
	3	2	2-3-2021	0.81	1.51	6.70	31.20	42.90	23.30	75	531			3.69		13.12
		3	29-3-2021	0.81	1.50	6.18	17.70	41.90	37.80	25	405		6.97	2.73		41.27
		1	30-11-2018	0.88	1.71	7.54	9.20	49.30	38.60	11	221					
	1	2	4-1-2019	0.88	1.76	7.36	5.50	41.40	50.10	25	325					13.91
		3	18-3-2019	0.88	1.71	7.35	11.70	55.50	29.90	25	592				51.73	37.03
		1	2-12-2019	0.81	1.87	7.03	17.00	38.60	41.20	13	529					134.77
S11	2	2	25-2-2020	0.81	1.71	6.86	11.90	26.60	58.60	50	546	7.06	174.52			
		3	24-3-2020	0.81	1.62	6.95	8.80	21.10	67.30	31	570	33.60	35.08			
		1	10-9-2020	0.88	1.97	7.39	15.60	40.10	40.90	65	291					
	3	2	26-1-2021	0.88	1.81	6.92	10.00	22.00	64.90	107	791		206.81			21.48
		3	25-3-2021	0.88	2.61	7.17	14.20	61.30	20.00	27	294		42.08	2.77		17.36
S12	3	1	23-9-2020	0.94	1.86	7.36	6.90	11.00	78.90	19	498					

		2	25-1-2021	0.94	1.78	7.07	6.00	9.20	81.70	13	532			1.86		26.31
		3	9-3-2021	0.94	1.99	7.49	5.40	12.60	78.60	38	820		42.92	2.67		21.14
S13	3	1	23-9-2020	0.88	1.47	7.42	19.80	61.20	16.50	38	556					
		2	27-1-2021	0.88	1.31	7.45	22.20	58.70	16.80	13	615		20.82			19.47
		3	23-3-2021	0.88	1.70	7.25	14.00	58.80	24.30	126	647			5.35		32.49
S14	1	1	5-12-2018	0.85	1.00	7.73	17.20	48.10	33.00	13	214					
		2	23-1-2019	0.85	1.24	7.48	24.00	48.20	25.70	11	890					6.95
		3	13-3-2019	0.85	0.94	7.37	20.90	49.50	28.00	19	559					
	2	1	19-11-2019	0.87	1.33	7.85	21.10	42.70	33.90	43	315					
		2	5-2-2020	0.87	1.63	7.58	20.00	38.60	38.60	13	352	10.96				
		3	24-3-2020	0.87	1.22	7.58	21.40	42.00	34.50	63	377	13.50	14.89			
S15	1	1	4-12-2018	0.87	1.29	8.00	12.70	70.70	14.40	27	459					
		2	22-1-2019	0.87	1.06	8.08	17.00	66.70	14.50	149	856					15.37
		3	13-3-2019	0.87	1.18	8.12	18.00	62.70	17.30	50	610				28.02	20.23
	2	1	19-11-2019	0.88	1.20	8.06	22.50	61.70	13.70	11	519					
		2	11-2-2020	0.88	1.64	7.94	25.10	56.30	15.80	50	426	14.40				
		3	24-3-2020	0.88	1.25	7.93	23.80	61.00	13.10	75	310	18.22			25.64	
S16	1	1	6-12-2018	0.82	1.06	7.72	27.90	54.50	15.80	15	75					
		2	1-2-2019	0.82	1.24	8.06	23.10	51.80	23.00	63	617					18.94
		3	25-3-2019	0.82	1.06	7.50	26.30	53.40	18.50	27	446					51.73
	2	1	4-10-2019	0.85	1.42	8.07	20.60	46.30	30.70	11	389					
		2	3-3-2020	0.85	1.24	7.77	13.90	37.50	46.50	57	142		27.76			
S17	2	1	3-12-2019	0.83	2.10	6.41	31.30	44.10	21.00	13	333					
		2	4-3-2020	0.83	1.85	6.39	25.00	46.60	25.20	38	165	20.37			8.16	
		3	25-3-2020	0.83	1.96	6.42	31.20	45.20	20.20	25	661	24.58	194.32	2.82	14.77	
	3	1	28-10-2020	0.83	2.13	6.47	21.20	48.90	26.20	36	409	53.62	132.92			
		2	3-2-2021	0.83	1.92	7.36	16.70	54.80	25.20	13	787			1.94		4.93

		3	15-4-2021	0.83	1.95	6.75	15.40	56.20	25.00	107	380			71.88		
S18	2	3	25-3-2020	0.74	1.92	6.43	33.90	42.10	20.70	38	600	19.77				
	3	1	14-10-2020	0.74	2.06	6.54	20.80	41.20	34.50	50	840	65.64				
S19org1	1	1	7-11-2018	0.83	1.65	7.10	12.10	49.10	36.00	31	243					
		2	15-11-2018	0.83	1.35	7.09	10.90	48.10	38.70	24	145					
		3	22-2-2019	0.83	1.29	7.43	18.10	57.50	22.20	13						
	2	1	3-12-2019	0.83	1.52	7.02	24.60	45.80	27.00	25	357					
		2	14-1-2020	0.83	1.42	7.19	29.00	52.60	16.00	50	511					
		3	23-3-2020	0.83	1.53	7.25	32.00	52.60	12.80	38	427				10.67	
	3	1	29-9-2020	0.83	1.55	7.21	29.40	55.60	12.30	31	641					
		3	18-2-2021	0.83	1.63	7.51	30.40	56.90	9.90	13	687					

Table S3: Properties of analyzed compounds taken from the pesticide properties database (PPDB)(11) including compound name, CAS number, pesticide type, chemical class and formula, molecular weight, water solubility, logarithmized octanol–water partition coefficient ($logK_{OW}$), the negative base–10 logarithm of the acid dissociation constant (pK_a), vapour pressure, logarithmized Freundlich soil organic carbon to water distribution coefficient ($logK_{f,OC}$), and the field soil degradation half–life ($DT_{50,field}$). Classifications of the vapour pressure, $logK_{f,OC}$ and the $DT_{50,field}$ are according to the PPDB(11) and indicated below the table.

No.	Compound Name	CAS No.	Type[c]	Chemical class	Chemical formula	Molecular weight [amu]	Water solubility at 20 ºC [mg/L]	$LogK_{OW}$ at 20 ºC, pH 7	pK_a at 25 ºC	Vapour pressure at 20 ºC [mPa]	$K_{f,oc}$ [mL/g]	$DT_{50,field}$ [days]
1	metribuzin DADK	52236-30-3	TP	of no. 9	$C_7H_{11}N_3O_2$	169.18	243	1.49		0.051	33	15.75[d]
2	atrazine desethyl	6190-65-4	TP	of no. 4	$C_6H_{10}ClN_5$	187.63	2700	1.51		12.44		45
3	2,6 dichlorobenzamid	2008-58-4	TP	of no. 25	$C_7H_5Cl_2NO$	190.03	1830	0.38	NI[e]	$2.0x10^{-2}$	40.98	137.7
4	atrazine	1912-24-9	H	triazine	$C_8H_{14}ClN_5$	215.68	35	2.7	1.7	0.039	174	29
5	clomazone	81777-89-1	H	oxazole	$C_{12}H_{14}ClNO_2$	239.7	1212	2.58	NI	27.0	128.3	27.3
6	chlorothalonil	1897-45-6	F	chloronitrile	$C_8Cl_4N_2$	265.91	0.81	2.94	NI	0.076	1288	17.9
7	metribuzin DA	35045-02-4	TP	of no. 9	$C_8H_{13}N_3OS$	265.9	475			$6.5x10^{-3}$	43.7	3.4[d]
8	pirimicarb	23103-98-2	I/A	carbamate	$C_{11}H_{18}N_4O_2$	238.39	3100	1.7	4.4	0.43	166.8	9
9	metribuzin	21087-64-9	H	triazinone	$C_8H_{14}N_4OS$	214.29	10700	1.7	1.3	0.121	48.3	19
10	ametryn	834-12-8	H	triazine	$C_9H_{17}N_5S$	227.12	200	2.63	10.07	0.365	5115	37
11	metalaxyl	57837-19-1	F	anilide/acryl-amino acid	$C_{15}H_{21}NO_4$	279.33	8400	1.75	0	0.75	162.3	14.1
12	prosulfocarb[a]	52888-80-9	H	thiocarbamate	$C_{14}H_{21}NOS$	251.39	13.2	4.48	NI	0.79	1693	9.8
13	S-metolachlor	87392-12-9	H	Chloro-acetamide	$C_{15}H_{22}ClNO_2$	283.79	480	3.05	NI	3.7	200.2	23.2
14	dicofol	115-32-2	I/A	Organo-chlorine	$C_{14}H_9Cl_5O$	370.49	0.8	4.3		0.25		80[d]
15	trifloxystrobin CGA 321113	252913-85-2	TP	of no. 23	$C_{19}H_{17}F_3N_2O_4$	394.0	21000			$5.50x10^{-3}$	116	70
16	triadimenol	55219-65-3	F	triazole	$C_{14}H_{18}ClN_3O_2$	295.76	72	3.18	NI	0.0005	273	36.5
17	α-endosulfane[b]	115-29-7	I/A	Organo-chlorine	$C_9H_6CL_6O_3S$	406.93	0.32	4.74		8.3		86
18	oxyfluorfen	42874-03-3	H	nitrophenol ether	$C_{15}H_{11}ClF_3NO_4$	361.7	0.116	4.86	NI	0.026	7566	73
19	fluazifop-p-butyl	69806-50-4	H	aryloxypheno-xypropionate	$C_{19}H_{20}F_3NO_4$	383.36	1	4.5		0.055		21[d]
20	cyproconazole	94361-06-5	F	triazole	$C_{15}H_{18}ClN_3O$	291.78	93	3.09	NI	0.026	364	129

21	β-endosulfane [b]	33213-65-9	I/A	Organo-chlorine	$C_9H_6Cl_6O_3S$	406.93	0.45	3.83	ND[f]		
22	carfentrazone-ethyl[a]	128639-02-1	H	triazolone	$C_{13}H_{14}Cl_2F_3N_3O_3$	412.19	29.3	3.7	NI	0.011	486.04	0.5
23	trifloxystrobin	141517-21-7	F	strobilurin	$C_{20}H_{19}F_3N_2O_4$	408.37	0.61	4.5	NI	$3.40x10^{-3}$	2287	1.7
24	benalaxyl	71626-11-4	F	fenilamine	$C_{20}H_{23}NO_3$	325.40	28.6	3.54	NI	0.572	555	66.8
25	fluopicolide	239110-15-7	F	benzamide	$C_{14}H_8C_{l3}F_3N_2O$	383.58	2.8	2.9	NI	$3.0x10^{-4}$	321.1	138.8
26	endosulfane sulphate	1031-07-8	TP	of no. 17 and 21	$C_9H_6Cl_6O_4S$	422.92	0.48	3.66				
27	tebuconazole	107534-96-3	F	triazole	$C_{16}H_{22}ClN_3O$	307.82	36	3.7	5.0	$1.30x10^{-3}$	769	47.1
28	epoxiconazole	135319-73-2	F	triazole	$C_{17}H_{13}ClFN_3O$	329.76	7.1	3.3	NI	$3.5x10^{-4}$	894	97.7
29	bifenthrin	82657-04-3	I/A	pyrethroid	$C_{23}H_{22}ClF_3O_2$	422.88	0.001	6.6	NI	0.0178		86.8
30	fenamidone	161326-34-7	F	imidazole	$C_{17}H_{17}N_3OS$	311.4	7.8	2.8	NI	0.00034	388	8.1
31	benthiavalicarb-isopropyl	177406-68-7	F	carbamate	$C_{18}H_{24}FN_3O_3S$	381.47	13.1	2.56	NI	0.3	180.6	13.8[d]
32	fenamidone RPA 410193	332855-88-6	TP	of no. 30	$C_{16}H_{15}N_3O_2$	281.31						
33	pyraclostrobin	175013-18-0	F	strobilurin	$C_{19}H_{18}ClN_3O_4$	387.82	1.9	3.99	NI	$2.6x10^{-5}$	9315	33.3
34	spirotetramat	203313-25-1	I/A	tetramic acid	$C_{21}H_{27}NO_5$	373.48	29.9	2.51	10.7	$5.6x10^{-6}$	281	0.7
35	boscalid	188425-85-6	F	carboxamide	$C_{18}H_{12}Cl_2N_2O$	343.21	4.6	2.96	NI	0.00072	772	254
36	deltamethrin	52918-63-5	I/A	pyrethroide	$C_{22}H_{19}Br_2NO_3$	505.20	0.0002	4.60	NI	0.0011	10240000	21
37	azoxystrobin	131860-33-8	F	strobilurin	$C_{22}H_{17}N_3O_5$	403.4	6.7	2.5	NI	$1.1x10^{-7}$	423	180.7
38	dimethomorph	110488-70-5	F	morpholine	$C_{21}H_{22}ClNO_4$	387.86	28.95	2.68	-1.3	$9.7x10^{-4}$	419.4	44

[a] not used or approved in Cuba according to the Official List of Pesticides of the Cuban Republic (2016)(12)[b] Compounds banned in Cuba since 2013(13) but appear in the Official List of Pesticides of the Cuban Republic(12)

[c] Type of pesticide: F: fungicide, H: herbicide, I/A: insecticide/acaricide, TP: transformation product

[d] $DT_{50,\ field}$ not available and the $DT_{50,\ typical}$ was put instead(11)

[e] NI: non-ionizable substance

[f] No pK_a identified

Vapour pressure (mPa): $<1x10^{-8}$ = low volatility, $> 1x10^{-3}$= high volatility

Log $K_{f,oc}$: < 1.18 = very mobile, 1.18 – 1.88 = mobile, 1.88 – 2.70 = moderately mobile, 2.70 – 3.60 = slightly mobile, > 3.60 = non–mobile

DT_{50}: <30 days = non-persistent, 30 – 100 days = moderately persistent, 100 – 365 days = persistent, > 365 days = very persistent

Empty cells indicate information not available in PPDB (2023)(11).

Table S4: Non–linear mixed effect (nlme) model of the sum of different compound residues found in soil. Results are shown in the tables below.

**Model**	$y_{i,j,k} = \mu + \alpha_i + \beta_j + \pi_k + \delta_{i,j,k} + \varepsilon_{i,j,k}$	
	Variable	Meaning
y	Dependent variable	Soil concentrations found (transformation: $Log_{10}(C_{soil})$ [µg/kg$_{dry\ weight\ (dw)}$]
µ	Grand mean	
α_i	Independent variable, fixed effect	Cultivation period (CP1 – 4), i = 1, 2, 3, 4
β_j	Independent variable, fixed effect	Sampling time (s1 – s3), j = 1, 2, 3
π_k	Independent variable, fixed effect	Type of residue (sum of fungicides (F), sum of herbicides (H), sum of insecticides (I), sum of transformation products (TP)), k = 1, 2, 3, 4
$\delta_{i,j,k}$	Independent variable, random effect	Deviation of site at the ith CP, j^{th} sampling time, and k^{th} type of residue
$\varepsilon_{i,j,k}$	Error	Error of split unit

**Influence of cultivation period, sampling time, type of pesticides on logarithmized concentration detected in soil sample**			
	**p-value**		
CP[a]	0.0038**		
Sampling time[b]	<0.0001***		
Type of pesticide[c]	<0.0001***		
Signif. codes:	<0.001***<br><0.01**<br>≤0.05*		
[a]: cultivation period: 2018-2019 (CP1), 2019-2020 (CP2), 2020-2021 (CP3), 2021-2022 (CP4).<br>[b]: sampling time: before planting potato (s1), peak pesticide application period (s2) and harvest (s3).<br>[c]: type of pesticide: fungicide (F), herbicide (H), insecticide (I), transformation product (TP)			
**Pairwise comparisons of <u>cultivation period</u>**			
**p-value**	**CP1**	**CP2**	**CP3**
**CP2**	1.0	-	-
**CP3**	1.0	1.0	-
**CP4**	1.0	1.0	0.071
**Pairwise comparisons of <u>sampling time</u>**			
**p-value**	**s1**	**s2**	
**s2**	5.4e-10***	-	
**s3**	8.6e-11***	1.0	
**Pairwise comparisons of <u>type of residues</u>**			
**p-value**	**F**	**H**	**I**
**H**	1.0	-	-
**I**	<2e-16***	<2e-16***	-
**TP**	3.8e-05***	0.0024**	1.1e-08***

Table S5: Non–linear mixed effect (nlme) model of the residues of azoxystrobin (fungicide), dicofol (insecticide), dimethomorph (herbicide), S-metolachlor (herbicide), and trifloxystrobin CGA (transformation product of trifloxystrobin) found in soil. Results are shown in the tables below. If sampling time was not significant, no pairwise comparison was listed.

**Model**	$y_{i,j,k} = \mu + \alpha_i + \delta_i + \varepsilon_i$	
	Variable	Meaning
y	Dependent variable	Soil concentrations found (transformation: $Log_{10}(C_{soil})$, [$\mu g/kg_{dry\ weight\ (dw)}$]
μ	Grand mean	
α_i	Independent variable, fixed effect	Sampling time (s1 – s3), i = 1, 2, 3
$\delta_{i,j}$	Independent variable, random effect	Deviation of site at the ith sampling time
ε_i	Error	Error of split unit

**Influence of sampling time on azoxystrobin concentrations detected in soil samples**		
**p-value**	0.0368	
	**s1**	**s2**
**s2**	1.0	-
**s3**	0.049	0.258
**Influence of sampling time on dicofol concentrations detected in soil samples**		
**p-value**	0.8609	
**Influence of sampling time on dimethomorph concentrations detected in soil samples**		
**p-value**	0.0556	
**Influence of sampling time on S-metolachlor concentrations detected in soil samples**		
**p-value**	<0.0001	
	**s1**	**s2**
**s2**	2.5e-06	-
**s3**	0.00029	0.10144
**Influence of sampling time on trifloxystrobin CGA concentrations detected in soil samples**		
**p-value**	0.0042	
	**s1**	**s2**
**s2**	0.2118	-
**s3**	0.0011	0.2737

Table S6: Effect data for earthworms (*E. fetida*) such as lethal concentration that kills half of the tested population after 14 days (acute LC_{50}), the chronic no–observed effect concentration (NOEC) for reproduction, the predicted non–effect concentration (PNEC) or effect concentration (EC) from the pesticides properties database (PPDB)(11) and analyzed compounds are listed below. For the establishment of the assessment factor (AF) values please see main text.

No.	Compound Name	Earthworms – acute LC_{50}	Earthworms – chronic NOEC, reproduction	PNEC or EC	AF
		(mg/kg)			
1	metribuzin DADK[a]	> 1000	> 100	1	10
2	atrazine desethyl[a]	n.a. [c]	n.a.	0.01	1000
3	2,6 dichlorobenzamid[a]	> 750	n.a.	0.08	1000
4	atrazine	79	n.a.	0.08	1000
5	clomazone[b]	78	> 0.8	n.a.	10
6	chlorothalonil	268.5	50	5.00	10
7	metribuzin DA[a]	n.a.	≥ 50	0.50	10
8	pirimicarb[b]	653	5.46		10
9	metribuzin	427	> 52.3	5.23	10
10	ametryn	166	n.a.	0.16	10
11	metalaxyl	> 1000	40	4.00	10
12	prosulfocarb	71.8	n.a.	0.07	1000
13	S-metolachlor	570	<26.65	2.67	10
14	dicofol	43.1	n.a.	0.04	1000
15	trifloxystrobin CGA[a]	> 1000	n.a.	0.10	1000
16	triadimenol	> 390.5	n.a.	0.39	1000
17	α-endosulfane	14	n.a.	0.01	1000
18	oxyfluorfen	> 500	12.0	1.20	10
19	fluazifop-p-butyl[b]	> 500	n.a.		1000
20	cyproconazole	168	0.75	0.08	10
21	β-endosulfane	14	n.a.	0.01	1000
22	carfentrazone-ethyl[a]	> 410	17.72	1.77	10
23	trifloxystrobin	> 500	3.5	0.35	10
24	benalaxyl	180	36.4	3.64	10
25	fluopicolide	> 500	62.5	6.25	10
26	endosulfane sulphate[b]	n.a.	n.a.		
27	tebuconazole	1381	10	1.00	10
28	epoxiconazole	> 500	≥ 3.24	0.32	10
29	bifenthrin	> 8.0	1.065	0.11	10
30	fenamidone[b]	> 25	0.315		10
31	benthiavalicarb-isopropyl	> 500	162.0	16.20	10
32	fenamidone RPA[a]	> 25	0.315	0.003	10
33	pyraclostrobin	567	23.1	2.31	10
34	spirotetramat	> 1000	100	10.00	10
35	boscalid	> 500	1.197	0.12	10

36	deltamethrin[b]	> 645	0.165		10
37	azoxystrobin	28.3	3	0.30	10
38	dimethomorph	> 500	60	6.00	10

[a] when no NOEC for a transformation product was available, the NOEC of the parent compound was taken and divided by 10 according to Vašíčková et al.(14) Specifically, according to the PPDB, (11) atrazine for *E. fetida* had only LC_{50} data. For the calculation of the PNEC of atrazine-desethyl, LC_{50} of atrazine was divided by 10.
[b] compound not detected, hence, no PNEC value
[c] n.a.: not available

Table S7: Pesticides applied (insecticide (I), herbicide (H), fungicide (F) to preceding crops grown at potato fields between cultivation periods (2018–2021).

Pesticides applied in potato	Type	Possible preceding crops
acetamiprid	I	Citrus fruits, tomato
acephate	I	Cabbage, tobacco, coffee, corn
abamectin	I	Citrus fruits, onion, tomato
ametryn	H	Sweet potato, taro, yucca, sugar cane, citrus fruits, pineapple, banana
amitraz	I	Citrus fruits, cabbage, potato, pepper
azoxystrobin	F	Rice, banaba (*Lagerstroemia speciosa*), banana, papaya, tobacco, vegetables such as potato, tomato, soybean
benalaxyl	F	Vegetables such as potato, pineapple
bentazone	H	Rice
bifenthrin	I	Rice, citrus fruits, beans, tomatoes, indoor crops, corn, potato, soybeans, tobacco, forestry
cyproconazole	F	Rice, citrus fruits, garlic, onion, tomato, beans, mango, potato, soybean
chlorphenapir	I	Citrus fruits, beans, tomato, cabbage
chlorothalonil	F	Garlic, onion, ornamentals, tomato, banana
diaphentiuron	I	Beans, vegetables
dimethomorph	F	Soybeans, tobacco, vegetables
diquat	H	Avocado, cocoa, coffee, citrus fruits, guava, mango, ornamentals, banana, sugar cane
epoxiconazole	F	Banana, rice
fenpiroxymate	I	Citrus fruits, bean, pepper, pineapple
fenoxaprop-p-ethyl	H	Rice, beans, vegetables such as onion, tomato, soybean
fipronil	I	Rice, citrus fruits, potato, pepper, tomato, forestry.
fluazifop-p-buthyl	H	Sugarcane, citrus fruits, beans, garlic, onions, peppers, tomatoes, bananas, soybeans, tobacco, papaya, guava, mango, pineapple
fluopicolide	F	Vegetables, pineapple, tobacco
fosetil-al	F	Citrus fruits, tomato, pineapple, tobacco, avocado
glyphosate	H	Rice, coffee, citrus fruits, pineapple, banana, sugarcane
ammonium glufosinate	H	Rice, coffee, sugarcane, citrus fruits, pasture, banana, coffee
imidacloprid	I	Rice, eggplant, cucurbits, peppers, tomatoes, beans, corn, soybeans, citrus fruits, papaya, tobacco
isoxaflutole	H	Sweet potato, taro, banana, yucca, sugarcane, garlic, onion, tomato, corn, pineapple
malathion	I	Avocado, cocoa, coffee, citrus fruits, guava, mango, rice, garlic, eggplant, pumpkin, onion, melon, cucumber, pepper, tomato, sweet potato, cabbage, cassava, forestry, beans, pasture, papaya, corn, millet, banana, pineapple, tobacco
mancozeb	F	Citrus fruits, tomato, beans, banana, tobacco
metribuzin	H	Sugar cane, soy beans, tomato
copper oxychloride	F	Avocado,papaya, mango, coffee, cocoa, citrus fruits, garlic, eggplant, onions, cabbage, cucurbits, beans, peppers, tomatoes, beets, carrot, forestry, beans, peanuts, banana, tobacco
pyraclostrobin	F	Sugar cane, citrus fruits, garlic, tobacco, beans, soybeans
propaquizafop	H	Garlic, onion, sugarcane, citrus fruits, cabbage, beans, peanuts,

		potato, banana, beet, soybean, tomato, carrot
propamocarb hydrochloride	F	Tobacco, citrus fruits, vegetables, pineapple
propineb	F	Vegetables, tobacco
S-metalochlor	H	Sugar cane, beans, corn, soybean
spirodichlofen	I	Citrus fruits, tomato, banana
spiromesiphen	I	Vegetables
tebuconazole	F	Garlic, onion, rice, tomato, banana
terbutrin	H	Sugar cane, corn, sorghum
tetraconazol	F	Coffee, beans, corn, soybeans, tomato, rice, banana
triadimenol	F	Coffee, banana, citrus fruits, beans, pump fruit, tomato, pineapple, soybeans, rice
trifloxystrobin	F	Rice, citrus fruits, garlic, onion, tomato, beans, mango, soybeans
valiphenalate	F	Tobacco

Table S8: Observed half–lives ($DT_{50,obs}$) of Figure 2. Values in parentheses are without outliers. For ametryn and S-metolachlor s1 values were considered outliers, for azoxystrobin all s1 values and the half–life of 138 days, for chlorothalonil the half–life of 72 days, and for cyproconazole none.

Compound	Ametryn	Azoxy-strobin	Chloro-thalonil	Cypro-conazole	S-metola-chlor
**Min**	7.0 (7.0)	4.8 (4.8)	0.7 (0.7)	8.2	4.9 (4.9)
**1st quantile**	11 (11)	15 (14)	2.6 (2.5)	12	16 (15)
**Median over all sites**	18 (15)	35 (27)	2.8 (2.8)	17	21 (20)
**Geom[a] mean over all sites**	20 (15)	32 (23)	3.6 (3.1)	19	21 (18)
**Median[b] over all sites from geom mean/site**	**16**	**34**	**3.3**	**16**	**23**
**3rd quantile**	25 (20)	62 (38)	5.1 (4.9)	27	31 (25)
**Max**	81 (34)	138 (74)	72 (6.0)	52	135 (52)
**PPDB[11,c]** (dotted line in Figure 2)	37	181	18	129	23

[a] geom: geometric
[b] $DT_{50,median}$ in text and dashed line in Figure 2
[c] PPDB:(11) Pesticides properties database

Table S9: Sensitivity analysis of $DT_{50,obs}$ by varying soil depth influencing the predicted environmental concentration (A_0, eq.2, eq.3a to 3d). The crop interception was varied on the upper limit because the fungicides were normally applied between 50–89 days after planting, which include vegetative growth, tuber initiation and bulking (30–60 days), maturation and ev. vine yellowing (60 days to final).(15)

**Compound**	**$DT_{50,obs}$ (d) at soil depth of:**				**crop interception f_{int}**	
**Ametryn**	**20 cm**	**5 cm**	**10 cm**	**40 cm**	**n.a.[a]**	
Min	7.0	3.8	5.0	10		
1st quantile	11	8.7	9.8	13		
**Median over all sites**	**18**	**14**	**15**	**19**		
3rd quantile	25	25	25	31		
Max	81	81	81	81		
**Azoxystrobin**	**20 cm**	**5 cm**	**10 cm**	**40 cm**	**0.4**	**0.8**
Min	4.8	1.0	1.9	n.a.	4.4	n.a.
1st quantile	15	10	12	15	14	11
**Median over all sites**	**35**	**15**	**21**	**39**	**29**	**35**
3rd quantile	62	51	51	76	71	71
Max	138	138	138	207	138	184
**Chlorothalonil**	**20 cm**	**5 cm**	**10 cm**	**40 cm**	**0.4**	**0.8**
Min	0.7	0.5	0.6	n.a.	0.6	n.a.
1st quantile	2.6	1.8	2.1	3.0	2.4	3.3
**Median over all sites**	**2.8**	**2.1**	**2.5**	**3.4**	**2.7**	**3.7**
3rd quantile	5.1	3.4	4.0	6.3	4.8	6.8
Max	72	72	72	72	72	72
**Cyproconazole**	**20 cm**	**5 cm**	**10 cm**	**40 cm**	**0.4**	**0.8**
Min	8.2	2.2	3.4	n.a.	6.0	n.a.
1st quantile	12	6.4	8.9	n.a.	11	n.a.
**Median over all sites**	**17**	**7.4**	**11**	**13**	**15**	**13**
3rd quantile	27	14	14	23	24	24
Max	52	52	52	52	52	55
**S-metolachlor**	**20 cm**	**5 cm**	**10 cm**	**40 cm**	**n.a.**	
Min	4.9	0.5	1.0	n.a.		
1st quantile	16	7.5	10	15		
**Median over all sites**	**21**	**15**	**17**	**25**		
3rd quantile	31	27	27	41		
Max	135	135	135	135		

[a] n.a.: not applicable because pre–emergence herbicide or negative value

Table S10: Chronic risk for earthworms (*Eisenia fetida*), as sum of risk quotients (ΣRQs[a]) in soils of 18 conventional potato production sites (S01 to S18), one organic potato production site (S19org1 and replicate S19org2) and two control sites (S20cont and S21cont) at three sampling times: before planting potato (s1), peak pesticide application (s2) and harvest (s3) over four consecutive cultivation periods: 2018–2019 (CP1), 2019–2020 (CP2), 2020–2021 (CP3), and 2021–2022 (CP4).

Site	CP1	CP2	CP3	CP4	
S01	0.34	0.4	0.19	0.15	s1
S02	0.29	0.42	0.22	0.44	
S03	0.16	0.17	0.17	0.17	
S04	*[b]	0.43	*	*	
S05	0.16	0.21	*	*	
S06	0.40	*	*	*	
S07	*	*	0.24	0.18	
S08	*	*	0.0005	0.36	
S09	0.07	0.01	0.1	0.11	
S10	0.1	0.11	0.07	0.08	
S11	0.16	0.16	0.06	0.06	
S012	*	*	0.06	0.12	
S013	*	*	0.002	0.08	
S14	0.20	0.20	*	*	
S15	0.09	0.04	*	*	
S16	0.44	0.50	*	*	
S17	*	*	0.15	0.09	
S18	*	[c]	0.20	*	
$S19_{org1}$	0.04.	*	*	0.03	
$S19_{org2}$	*	*	*	0.02	
$S20_{cont}$	*	*	*	0.08	
$S21_{cont}$	*	*	*	*	
S01	0.45	0.18	0.51	0.16	s2
S02	2.33	0.97	0.73	0.39	
S03	0.20	0.29	1.13	0.14	
S04	*		*	*	
S05	0.19	0.17	*	*	
S06		*	*	*	
S07	*	*	1.24	0.34	
S08	*	*	0.44	0.41	
S09	0.47	0.77	0.73	0.11	
S10	0.12	0.89	0.22	0.1	
S11	2.16	1.66	0.43	0.19	
S012	*	*	0.44	0.17	
S013	*	*	0.09	0.15	
S14	0.1	0.38	*	*	
S15	0.03	2.42	*	*	
S16	0.19	0.51	*	*	
S17	*	2.91	0.27	0.37	
S18	*			*	
$S19_{org1}$	*	*		0.02	
$S19_{org2}$	*	*	*	0.01	
$S20_{cont}$	*	*	*	*	

$S21_{cont}$	*	*	*	*	
S01	2.33	0.22	0.39	0.24	s3
S02	1.55	0.57	1.11	0.41	
S03	2.38	0.25	0.29	0.29	
S04	*	0.18	*	*	
S05	1.22	0.40	*	*	
S06	0.32	*	*	*	
S07	*	*	0.33	0.32	
S08	*	*	1.02	0.06	
S09	0.28	0.72	0.21	0.06	
S10	2.94	0.27	0.22	0.42	
S11	0.87	1.05	0.28	0.08	
S012	*	*	0.26	0.32	
S013	*	*	0.13	0.05	
S14	0.90	0.28	*	*	
S15	0.94	1.03	*	*	
S16	1.89		*	*	
S17	*	1.73	0.01	1.02	
S18	*	1.11		*	
$S19_{org1}$	*	0.03	0.38	0.01	
$S19_{org2}$	*	*	*	*	
$S20_{cont}$	*	*	*	*	
$S21_{cont}$	*	*	*	*	

[a] ΣRQs:
<0.01 negligible ecological risk, green
0.01 < ΣRQs < 0.1 low ecological risk, yellow
0.1 < ΣRQs < 1 medium ecological risk, orange
>1 high ecological risk, red
[b] concentrations below the limit of quantification acc. to Peña et al.(16)
[c] empty cells indicate no sampling

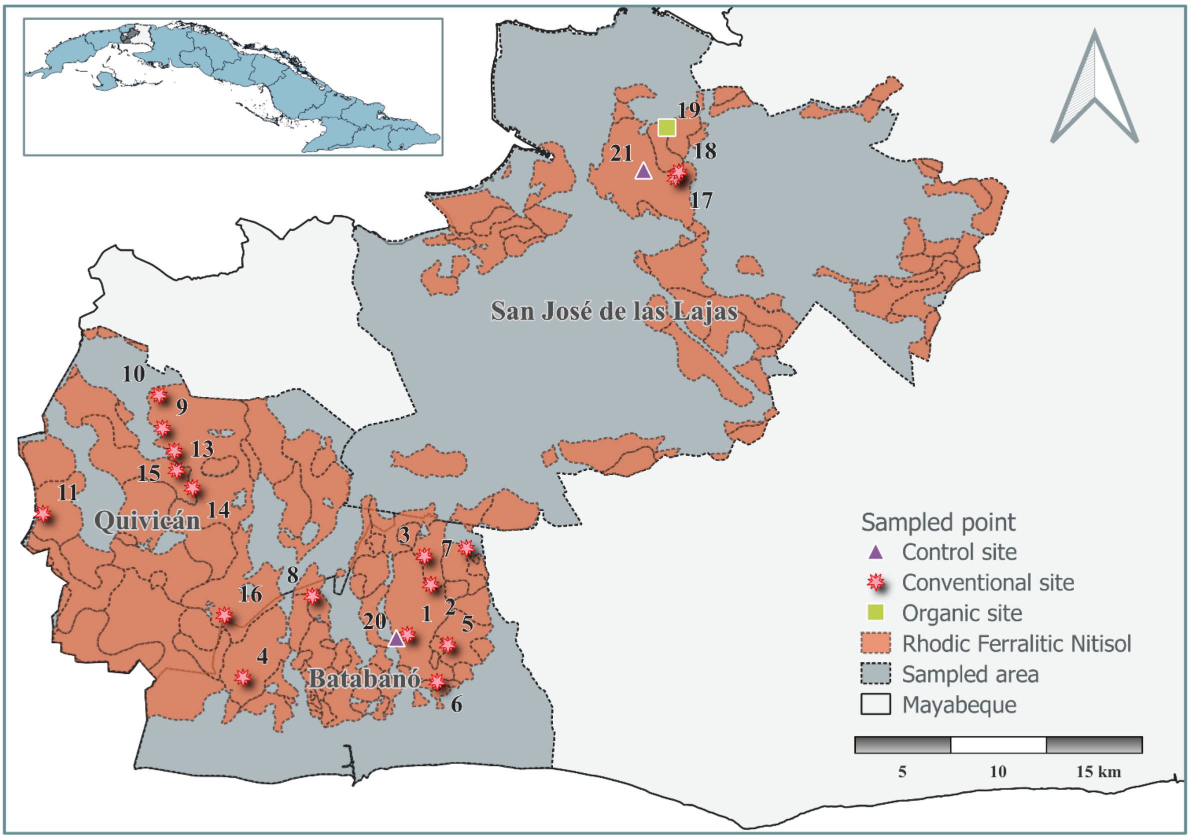

Figure S1: Investigated counties within the province of Mayabeque, Cuba (non-blue area in the top left corner) where soil samples were taken in Batabanó, Quivicán and San José de las Lajas (dark grey area). Individual soil sampling sites are marked with a red star (conventional sites, n=18), green square (organic site, n=1) and purple triangle (control site, n=2), and consecutively numbered (1–21). Sampling sites were georeferenced and soil layers visualized with the geographic information system.

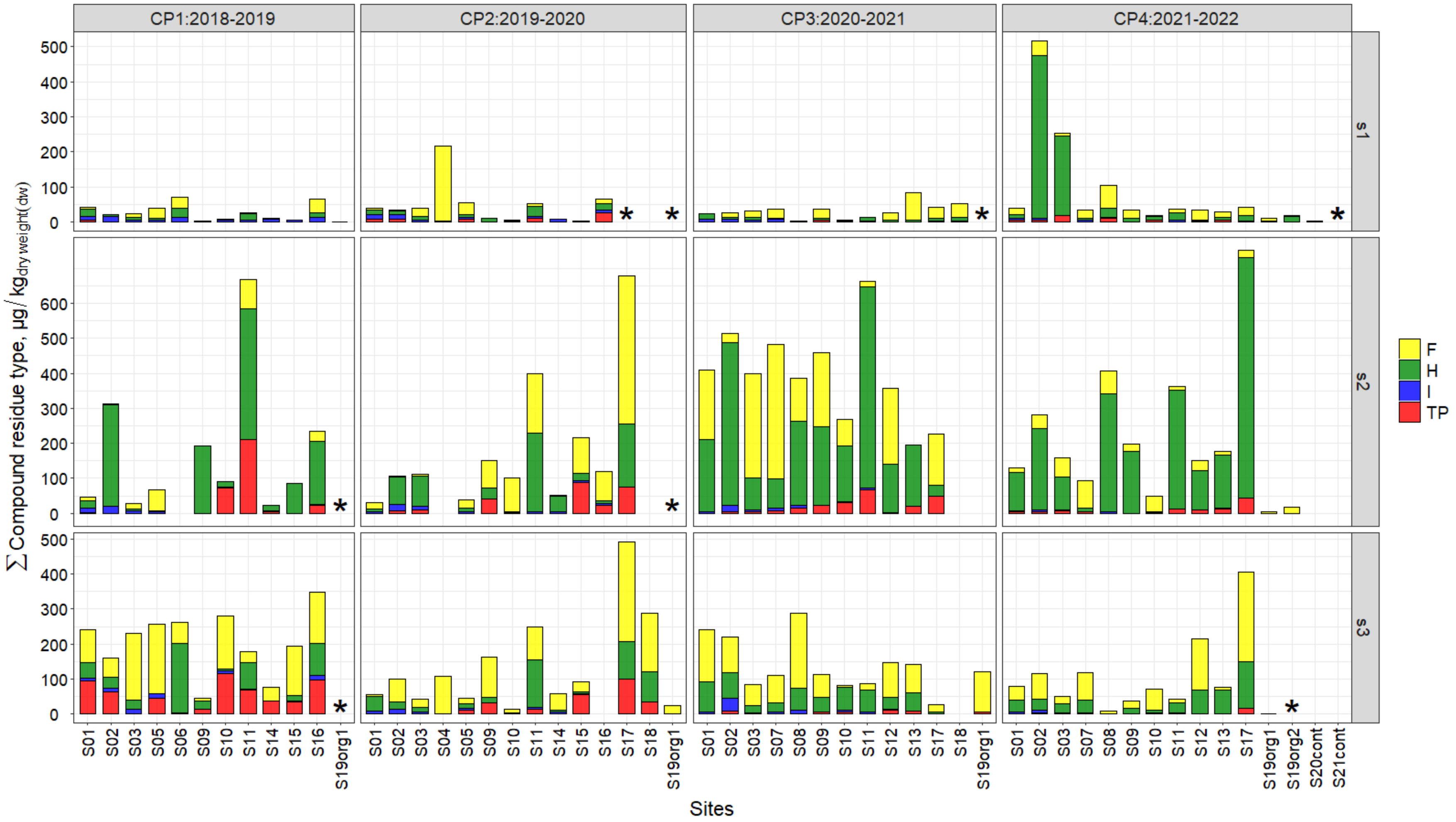

Figure S2: Concentrations of fungicides (F), herbicides (H), insecticides (I), and transformation products (TP) summed up per residue type (Σ compound residue type (µg/kg$_{\text{dry weight (dw)}}$)). Eighteen conventional potato production sites (S01 to S18) were sampled, one organically managed (S19$_{\text{org1,2}}$) and two control sites (S20$_{\text{cont}}$ and S21$_{\text{cont}}$), at three sampling times: before planting potato (s1), high pesticide application (s2) and harvest (s3) over four consecutive cultivation periods: 2018–2019 (CP1), 2019–2020 (CP2), 2020–2021 (CP3) and 2021–2022 (CP4). Cells with a star (*) represented concentrations below the limit of quantification and no samples were taken when cell is empty.

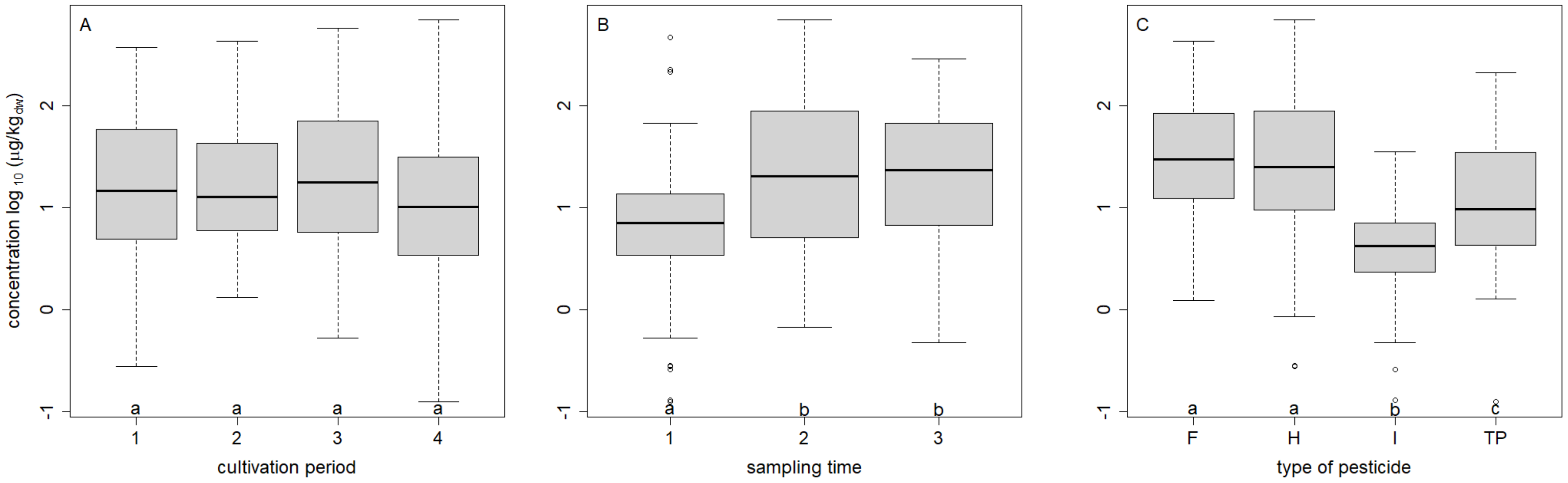

Figure S3: Boxplot between logarithmized concentrations of all analytes detected over four consecutive cultivation periods (A): 2018–2019 (1), 2019–2020 (2), 2020–2021 (3), and 2021–2022 (4), B: sampling times (before planting potato (1), peak pesticides application (2), and harvest (3)) and concerning C: type of compounds (fungicide (F), herbicide (H), insecticide (I), and transformation product (TP)). The boxes represent the 25th to 75th percentiles, the whiskers are the 10th and 90th percentiles, and the bold black line in the box indicates the median of the respective concentrations. Different letters indicate significate differences according to the evaluation with the non–linear mixed effect model (Table S4).

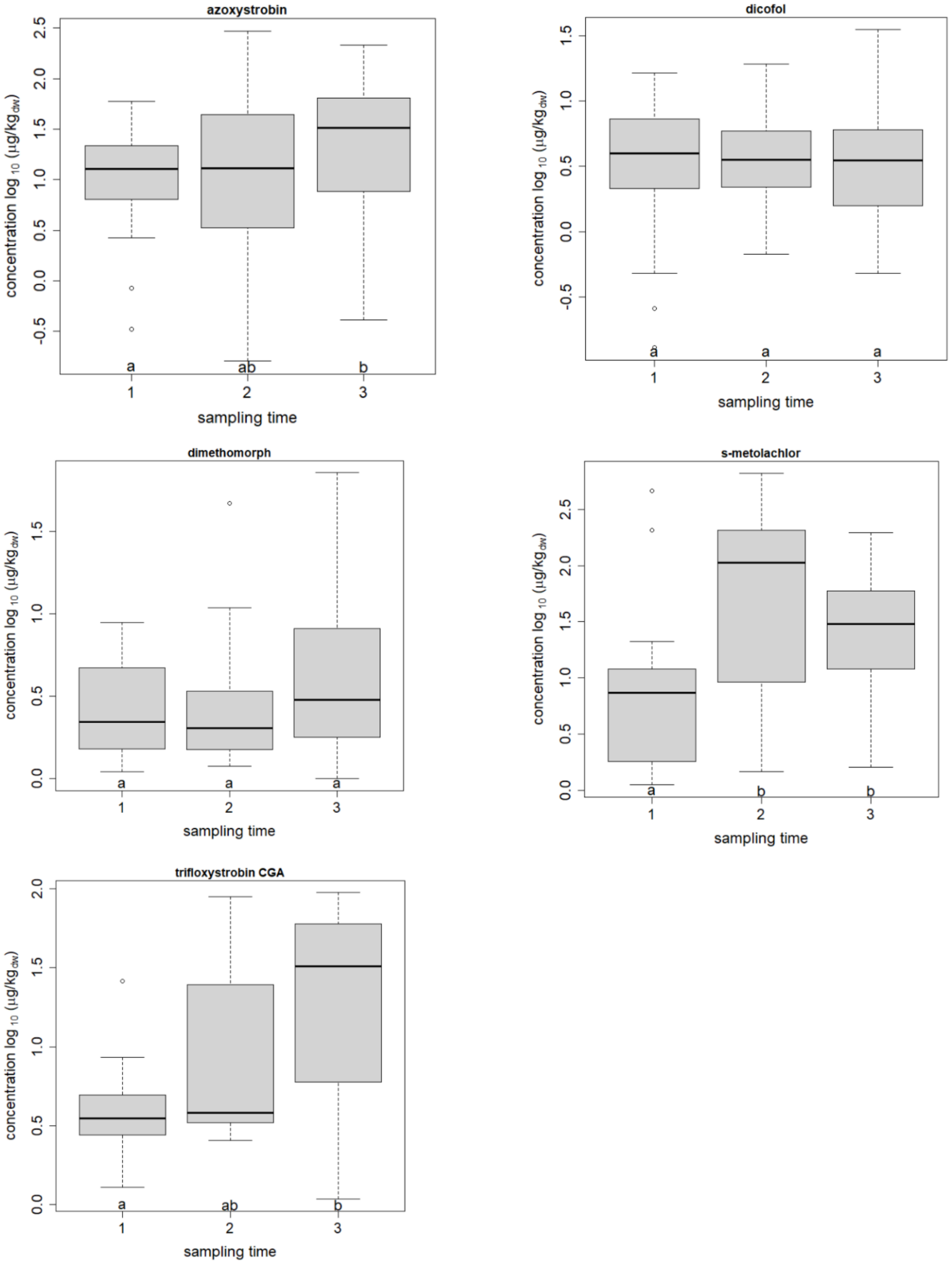

Figure S4: Boxplot of logarithmized concentrations of most frequent detected compounds and sampling time (before planting potato (1), peak pesticide application (2) and harvest (3)). The boxes represent the 25th to 75th percentiles, the whiskers are the 10th and 90th percentiles, the dots outliers, and the bold black line in the box indicates the median of the respective concentrations. Different letters indicate significate differences according to the evaluation with the non–linear mixed effect model (Table S5).

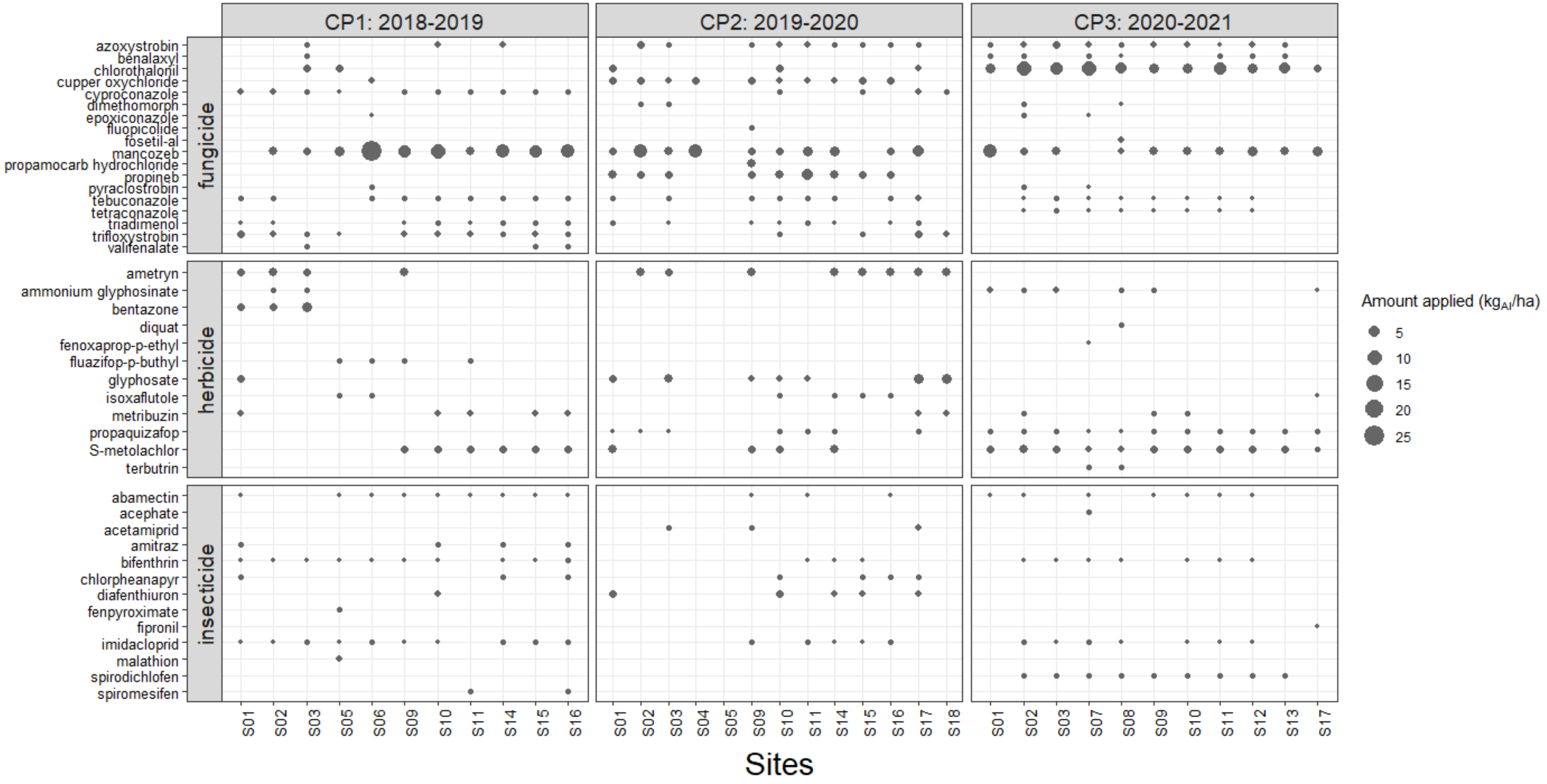

Figure S5: Active ingredients (AI) applied to 18 conventional potato production sites (S01 to S18), over three consecutive cultivation periods: 2018–2019 (CP1), 2019–2020 (CP2), and 2020–2021 (CP3). Points represent the sum of all dosages applied per compound (kg_{AI}/ha) per site and CP.

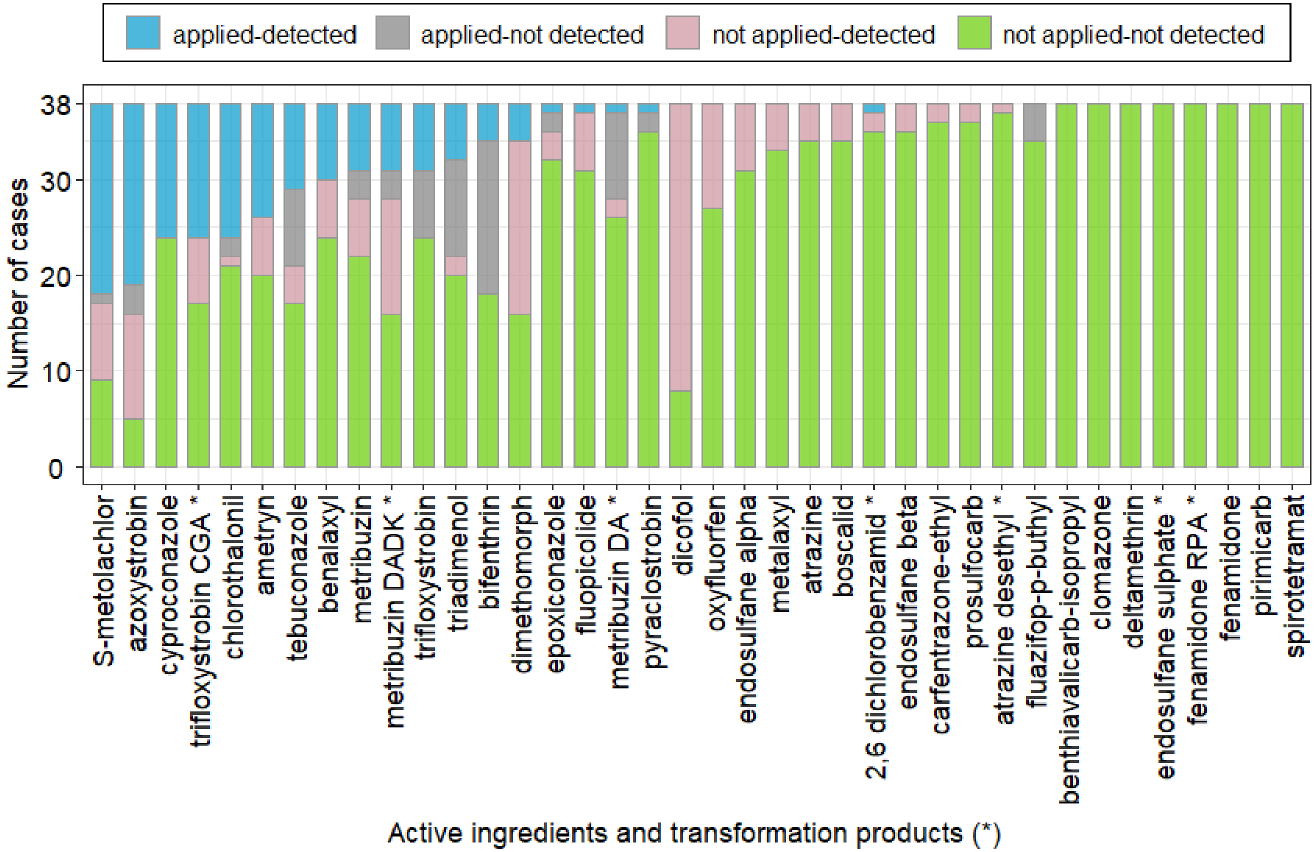

Figure S6: Relation between active ingredients (AIs) applied and detected including some transformation products (*) in soil samples. Pesticide applied and detected (true–positive, blue bar), pesticide applied and not detected (false–negative, grey bar), pesticide not applied and detected (false–positive, pink bar), pesticide not applied and not detected (true–negative, green bar) corresponded to 38 compounds at 38 sites (12 sites from cultivation period (CP)1, 14 from CP2 and 12 from CP3). Transformation products were related with the application of their parent AI.

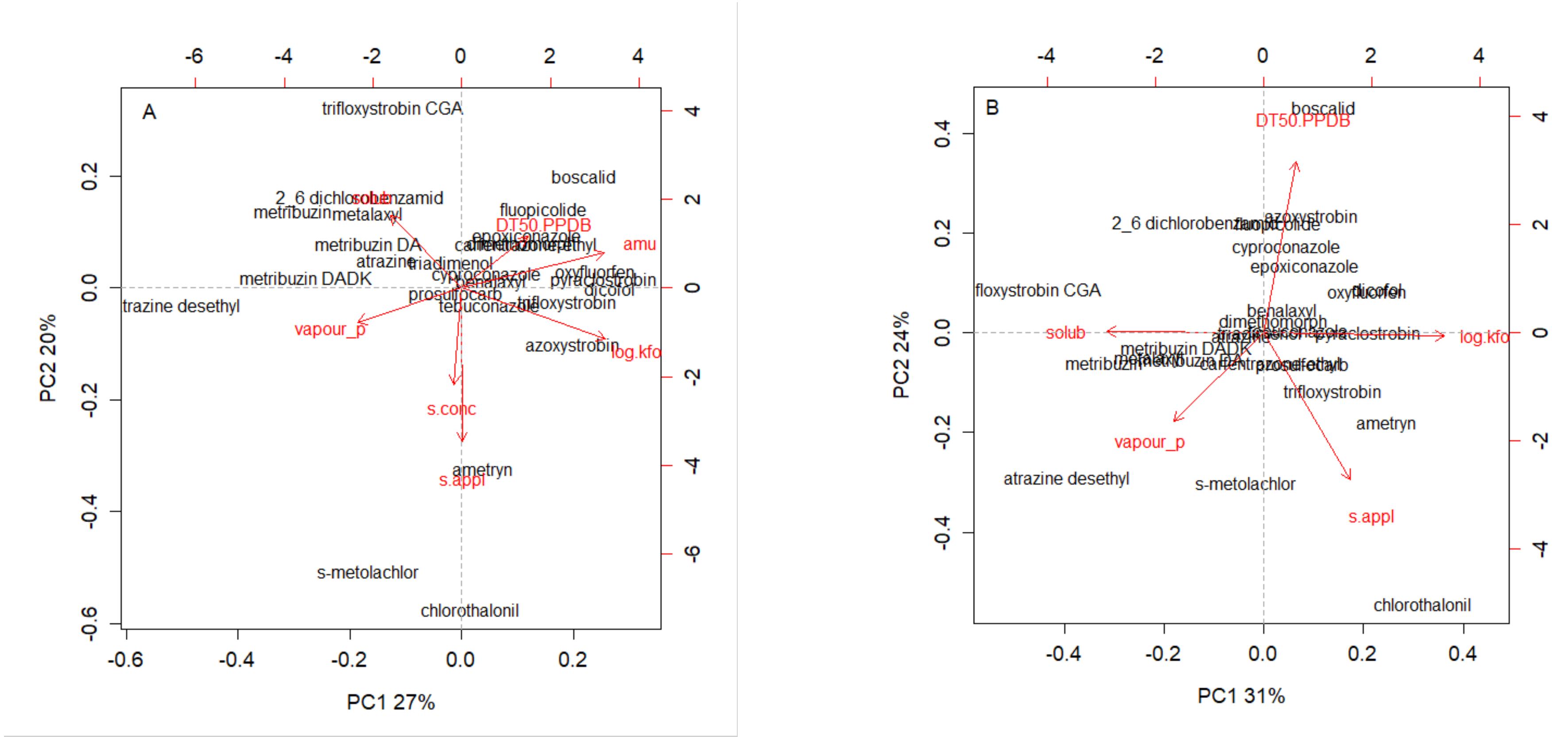

Figure S7: Principal component (PC) analysis of 28 compounds (observations in the biplot) analysed in this study. Arrows or vectors are the observation's physico–chemical properties (Table S3) such as (counter clockwise, panel A) logarithmized Freundlich water to organic carbon sorption coefficient (log K_{fOC}), molecular weight (amu), half-life ($DT_{50,PPDB}$), solubility (solub), vapour pressure (vapour_p), and environmental data such as the sum of applications (s.appl) and sum of residues in soil (s.conc). Percentage in the biplot indicate the loadings (%) of PC1 and PC2 and the corresponding axis the scores. The right y–axis and upper x–axis refer to the (Eigen)vectors. Panel B shows the optimized biplot where the molecular weight and the sum of residues in soil were removed due to bad representativeness and optimize the loadings.

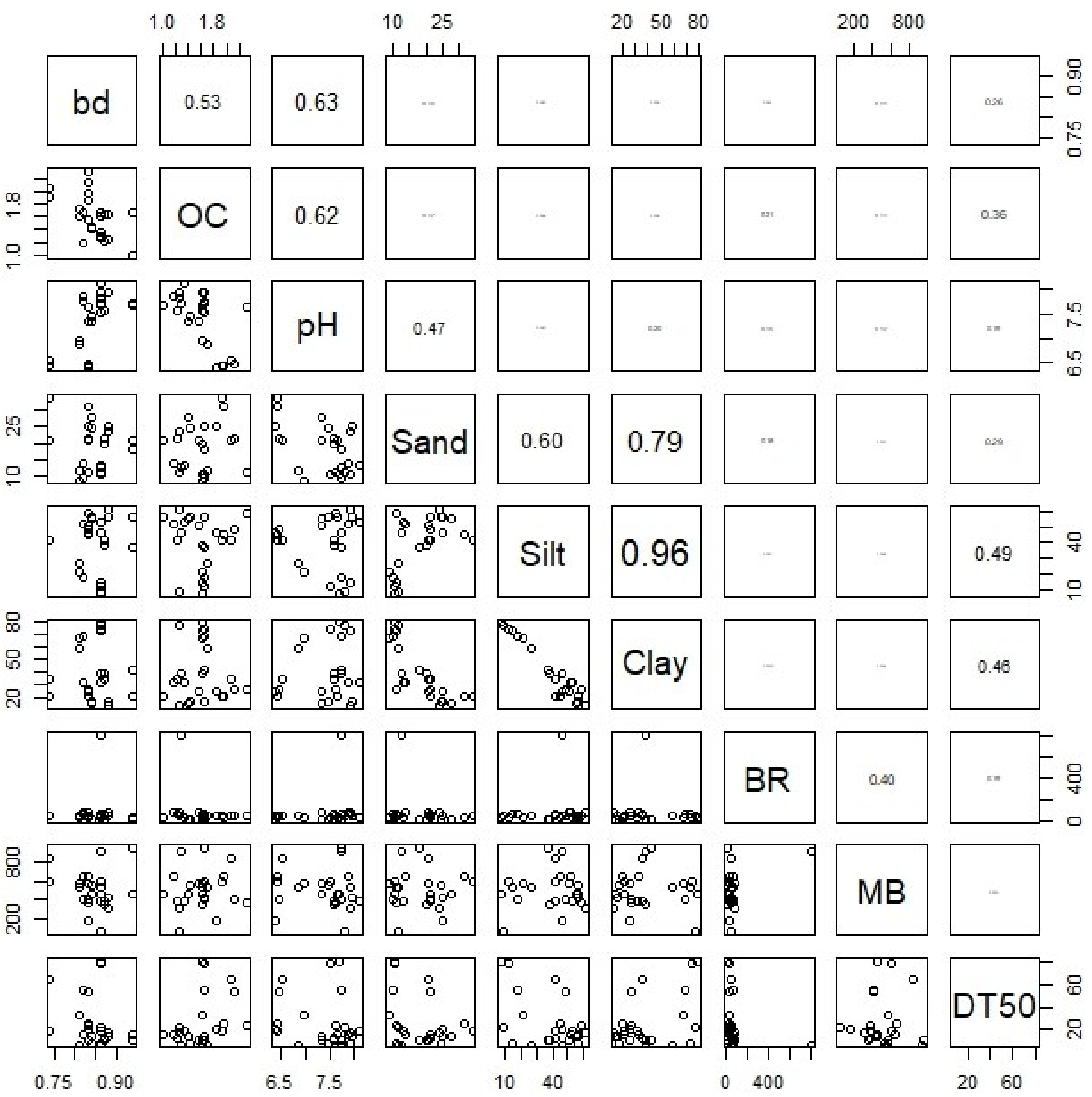

Figure S8: Correlations of soil properties (bulk density (bd, g/cm^3), organic carbon content (OC, %), pH (-), sand, silt and clay content (%), each, basal respiration (BR, $mg_{CO2}/(kg_{soil}*day)$), microbial biomass (MB, mg_C/kg_{soil}), and the observed, site specific half–lives (DT_{50}) of **ametryn**. The upper panels represent the absolute correlation coefficient and the size represents the value.

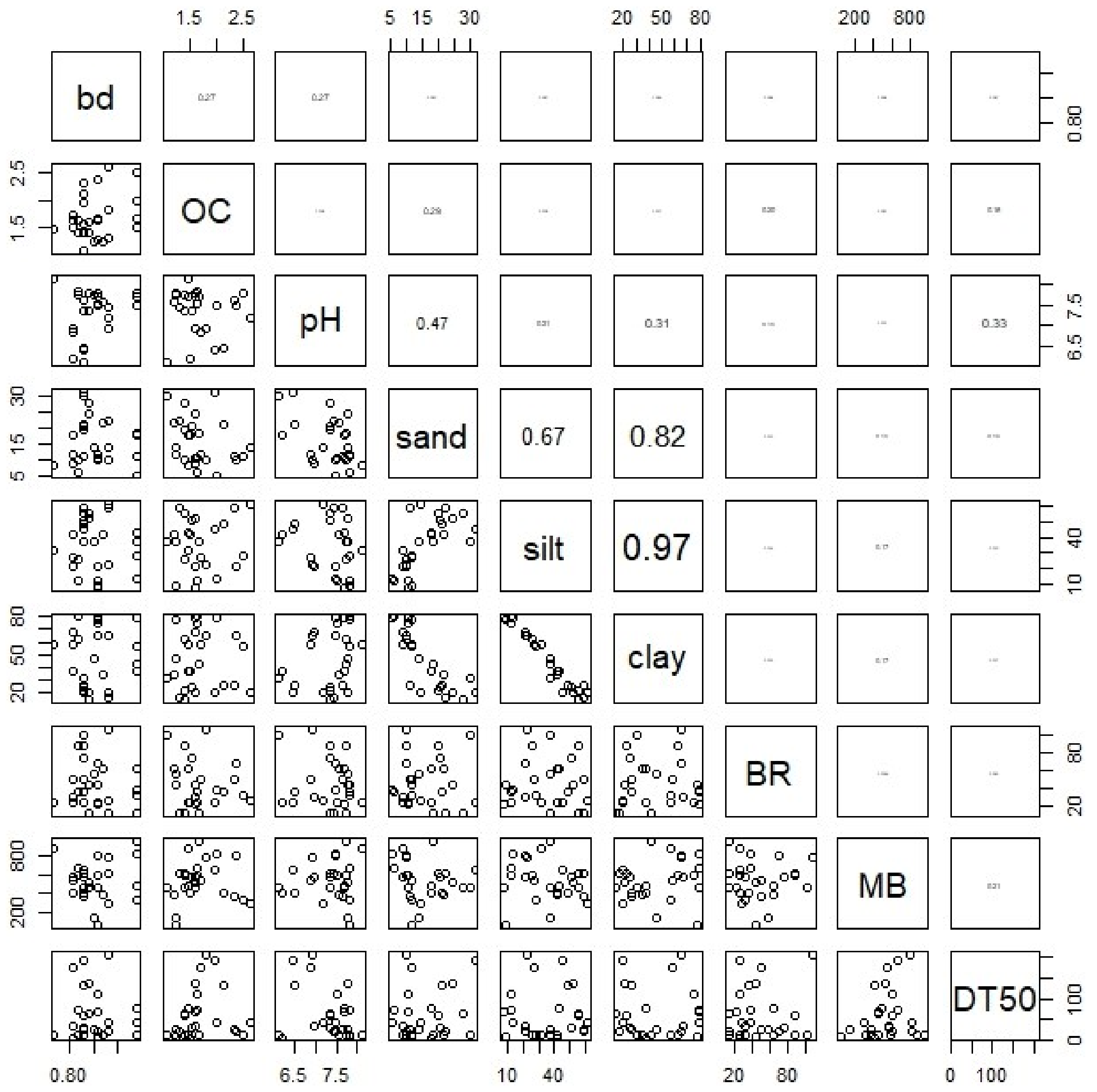

Figure S9: Correlations of soil properties (bulk density (bd, g/cm^3), organic carbon content (OC, %), pH (-), sand, silt and clay content (%), each, basal respiration (BR, $mg_{CO2}/(kg_{soil}*day)$), microbial biomass (MB, mg_C/kg_{soil}), and the observed, site specific half–lives (DT_{50}) of **azoxystrobin**. The upper panels represent the absolute correlation coefficient and the size represents the value.

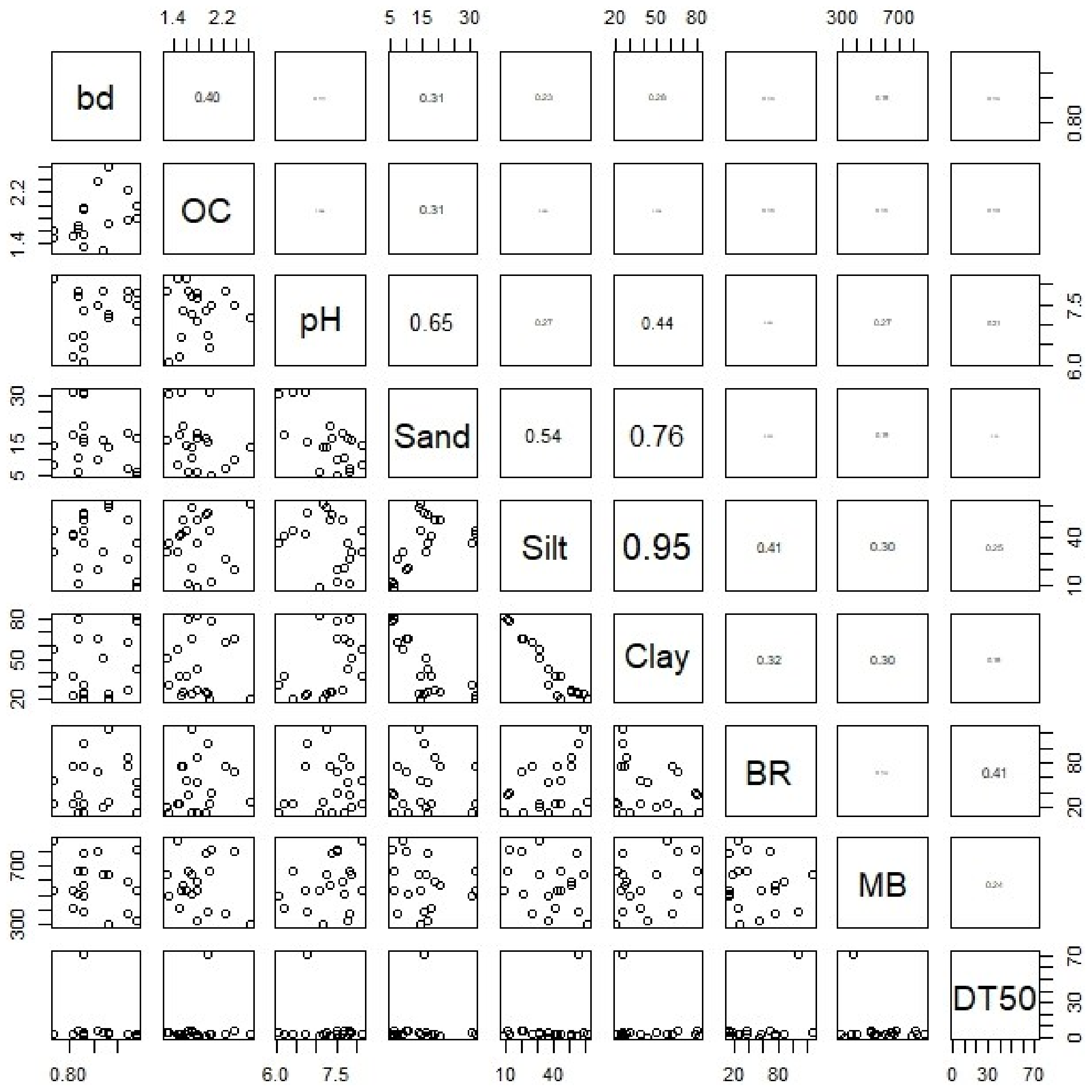

Figure S10: Correlations of soil properties (bulk density (bd, g/cm^3), organic carbon content (OC, %), pH (-), sand, silt and clay content (%), each, basal respiration (BR, $mg_{CO2}/(kg_{soil}*day)$), microbial biomass (MB, mg_C/kg_{soil}), and the observed, site specific half–lives (DT_{50}) of **chlorothalonil**. The upper panels represent the absolute correlation coefficient and the size represents the value.

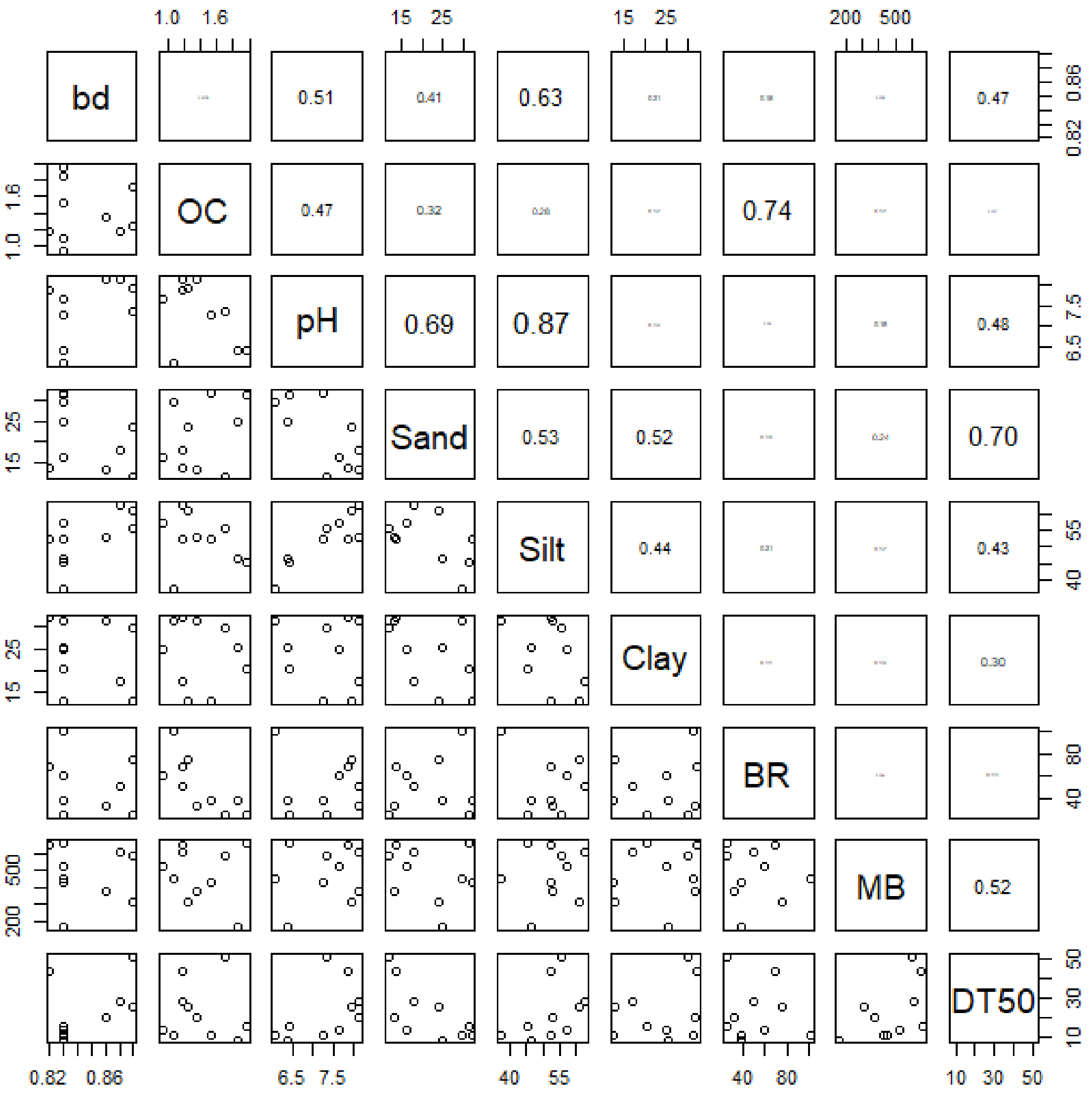

Figure S11: Correlations of soil properties (bulk density (bd, g/cm^3), organic carbon content (OC, %), pH (-), sand, silt and clay content (%), each, basal respiration (BR, $mg_{CO2}/(kg_{soil}*day)$), microbial biomass (MB, mg_C/kg_{soil}), and the observed, site specific half–lives (DT_{50}) of **cyproconazole**. The upper panels represent the absolute correlation coefficient and the size represents the value.

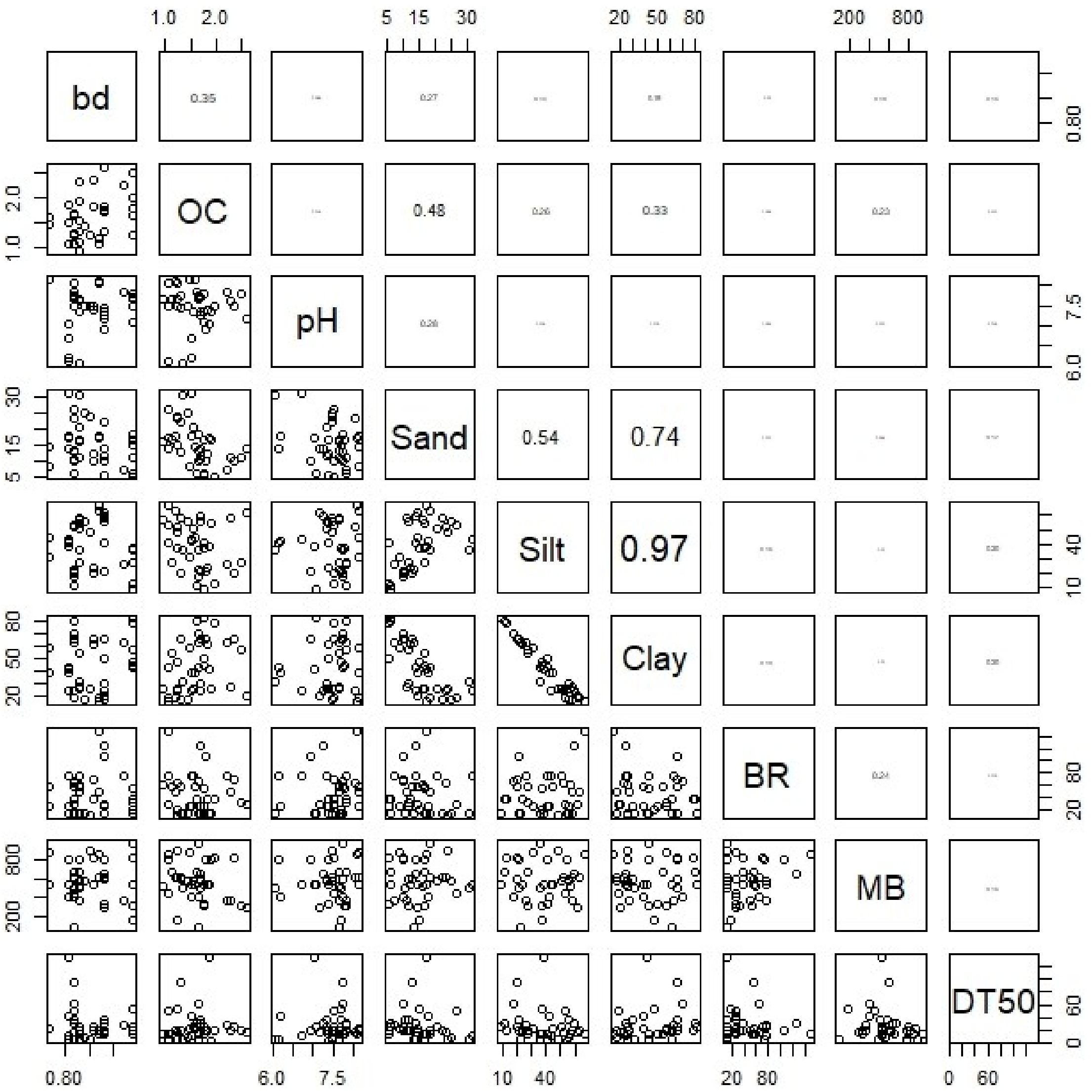

Figure S12: Correlations of soil properties (bulk density (bd, g/cm^3), organic carbon content (OC, %), pH (-), sand, silt and clay content (%), each, basal respiration (BR, $mg_{CO2}/(kg_{soil}*day)$), microbial biomass (MB, mg_C/kg_{soil}), and the observed, site specific half–life (DT_{50}) of **S-metolachlor**. The upper panels represent the absolute correlation coefficient and the size represents the value.

S3. References

1. FAL R. Schweizerische Referenzmethoden der Eidgenossischen Landwirtschaftlichen Forschungsanstalten. FAL-Eigenverlag, Zurich. 1996.

2. Anderson JP. Soil respiration. Methods of soil analysis: part 2 chemical and microbiological properties. 1983;9:831-71.

3. Vance ED, Brookes PC, Jenkinson DS. An extraction method for measuring soil microbial biomass C. Soil biology and Biochemistry. 1987;19(6):703-7.

4. Barroso Planas K. Tendencias en el uso de plaguicidas y agentes de control biológico en *Solanum tuberosum* L., en Cuba [Tesis de Maestría en Sanidad Vegetal]. San José de las Lajas: Universidad Agraria de La Habana "Fructuoso Rodríguez Pérez"; 2014.

5. Gallivan G, Surgeoner G, Kovach J. Pesticide risk reduction on crops in the province of Ontario. Journal of Environmental Quality. 2001;30(3):798-813.

6. Yanggen D, Crissman CC, Espinosa P. Los plaguicidas: impactos en producción, salud y medio ambiente en Carchi, Ecuador: Editorial Abya Yala; 2003.

7. Ramírez-Muñoz F, Fournier-Leiva ML, Ruepert C, Hidalgo-Ardón C. Uso de agroquímicos en el cultivo de papa en Pacayas, Cartago, Costa Rica. Agronomía Mesoamericana. 2014;25(2):339-45.

8. FAO. FAOSTAT Analytical Briefs, No 89 Rome.

9. Okonya JS, Kroschel J. A Cross-Sectional Study of Pesticide Use and Knowledge of Smallholder Potato Farmers in Uganda. Biomed Research International. 2015:9.

10. Anastassiadou M, Bellisai G, Bernasconi, G, Brancato A, Carrasco Cabrera L, Ferreira L, et al. Review of the existing maximum residue levels for cyproconazole according to Article 12 of Regulation (EC) No 396/2005 EFSA Journal. 2021;19:e06483.

11. PPDB. Pesticide properties database, agriculture & environment research unit (AERU), University of Hertfordshire, UK, https://sitem.herts.ac.uk/aeru/ppdb/en/atoz.htm [

12. MINAG. Lista Oficial de Plaguicidas Autorizados. Registro Central de Plaguicidas, República de Cuba. Ministerio de la Agricultura. 2016.

13. Pérez-Consuegra N, Montano-Pérez M. Los Plaguicidas Altamente Peligrosos en Cuba. IPEN/ACTAF/RAPAL Editora Agroecológica. 2021:56.

14. Vašíčková J, Hvězdová M, Kosubová P, Hofman J. Ecological risk assessment of pesticide residues in arable soils of the Czech Republic. Chemosphere. 2019;216:479-87.

15. MINAG. Instructivo Técnico para la producción de papa en Cuba. La Habana, Cuba: Ministerio de la Agricultura, Dirección de Agricultura, República de Cuba; 2016.

16. Peña B, Sosa D, Hilber I, Escobar A, Bucheli TD. Validation of a modified QuEChERS method for the quantification of residues of currently used pesticides in Cuban agricultural soils, using gas chromatography tandem mass spectrometry. Environmental Science and Pollution Research. 2024;31(23):33623-37.
